# Attitudes of nursing professionals in adverse events in neonatal and pediatric intensive care

**DOI:** 10.1590/1980-220X-REEUSP-2024-0407en

**Published:** 2025-07-11

**Authors:** Maria Salete de Brito Gomes, Ana Clara de Brito Gomes, Cynthia Lima Sampaio, Paulo César de Almeida, Jênifa Cavalcante dos Santos Santiago, Joselany Áfio Caetano, Roberta Meneses Oliveira

**Affiliations:** 1 Universidade de Fortaleza, Fortaleza, CE, Brazil.; 2 Universidade Federal do Ceará, Faculdade de Farmácia, Odontologia e Enfermagem, Departamento de Enfermagem, Fortaleza, CE, Brazil.; 3 Universidade Federal do Ceará, Departamento de Enfermagem, Programa de Pós-graduação, Fortaleza, CE, Brazil.; 4 Universidade Estadual do Ceará, Fortaleza, CE, Brazil.

**Keywords:** Nursing, Attitude of Health Personnel, Intensive Care Units, Neonatal, Patient Safety, Patient Harm, Enfermagem, Atitude do Pessoal de Saúde, Unidades de Terapia Intensiva Neonatal, Segurança do Paciente, Dano ao paciente

## Abstract

**Objective:**

To analyze the attitudes of nursing professionals towards structural and process factors that may predispose to adverse events in neonatal and pediatric intensive care units.

**Method:**

Cross-sectional study carried out in two public hospitals in Northeast Brazil in 2019. A sociodemographic/professional questionnaire and the Adverse Events Predisposition Scale were applied to 100 nursing professionals. The tests ANOVA, Tukey and of Student’s t were used to compare the means of the ideal and real scales, according to the sociodemographic variables, adopting p < 0.05.

**Results:**

Most professionals demonstrated low and medium perception of structure and process factors. A statistically significant association was found in the process dimension with: age group of 40–49 years, neonatal unit/hospital 2, and relation with a cooperative.

**Conclusion:**

Nursing professionals in neonatal/pediatric intensive care units have low and medium perception of factors predisposing to adverse events, which deserves investment from leaders to avoid that inadequate perceptions result in harm to patients.

## RESUMO

**Objetivo:**

Analisar atitudes de profissionais de enfermagem diante dos fatores de estrutura e processo que podem predispor a eventos adversos em unidades de terapia intensiva neonatal e pediátrica.

**Método:**

Estudo transversal realizado em dois hospitais públicos do Nordeste brasileiro em 2019. Aplicou-se questionário sociodemográfico/profissional e Escala de Predisposição a Eventos Adversos a 100 profissionais de enfermagem. Utilizaram-se os testes ANOVA, *Tukey* e t de *Student* para comparação das médias das escalas ideal e real, segundo as variáveis sociodemográficas, adotando-se p < 0,05.

**Resultados:**

A maioria dos profissionais demonstrou baixa e média percepção dos fatores de estrutura e processo. Constatou-se associação estatística significante na dimensão processo com: faixa etária 40–49 anos, unidade neonatal/hospital 2 e vínculo por cooperativa.

**Conclusão:**

Profissionais de enfermagem de unidades de terapias intensivas neonatal/pediátrica apresentam baixa e média percepção dos fatores predisponentes a eventos adversos, o que merece investimento de lideranças para evitar que percepções inadequadas resultem em dano aos pacientes.

## INTRODUCTION

A study carried out in the United States of America (USA) analyzed data from 244,542 adult patients hospitalized in 3,156 hospitals and observed a reduction in adverse event rates between 2010 and 2019. Despite this drop, the rates still remain high, with records of 139 adverse events per 1,000 patients with acute myocardial infarction, 116 for heart failure, 119 for pneumonia, and 130 for those undergoing major surgical procedures^(1)^. In Brazil, the incidence of adverse events was 4.4%, in a study carried out in emergency care, all of which were considered preventable^(2)^. These data highlight the magnitude of the problem and the need for in-depth analysis of the predisposition to events and investigation of strategies that minimize care risks, especially in intensive care environments, as they have peculiar characteristics that increase risks for patients, as is the case of pediatric and neonatal intensive care units (PICU and NICU). The complexity of the care provided to this clientele, the therapeutic arsenal, and decision-making in pediatric emergencies make the environment more conducive to the nursing team’s exhaustion. Given such vulnerability, the number of publications related to the safety of patients undergoing intensive care is increasing^(3,4,5)^.

Preventable adverse events include medication errors. Medication errors can be reduced by implementing several interventions using human systems-based approaches and technology. When it comes to reducing medication errors in the PICU and NICU, a multidisciplinary approach is paramount^(6)^.

Besides the multidisciplinary approach, a multilevel approach is required to reduce adverse events, according to a study on safety culture, which demonstrates that facilitating factors (organizational or management level activities) influence execution factors (frontline team actions) which, in turn, lead to patient safety results. However, the critical nature of psychological safety among nursing staff impacts this bottom line^(7)^. This way, the professional’s perspective and their perception of reality also deserve to be highlighted and investigated in more details.

The non-occurrence of adverse events has been an outcome indicator of the assessment of quality of care in intensive care^(8)^. However, not only the identification of adverse events, but also the perception of their risk can guide the development of corrective and preventive interventions, which is why it is essential to understand the perspective of the nursing professional so that approaches based on attitudes focused on structure and the work process can be formulated.

In view of the above, the objective was to analyze the attitudes of nursing professionals towards structural and process factors that may predispose to adverse events in NICU and PICU.

## METHOD

### Design of Study

Cross-sectional study

### Local

The study was carried out from January to April 2019, in two reference hospitals for pediatric and neonatal care, both specialized in high complexity, belonging to the public network of Ceará. Hospital 1, with 417 beds, is nationally recognized for its Cardiology and Pulmonology specialties. It has two pediatric intensive care units (PICU), totaling 17 beds, 8 for post-operative care and 9 for pre-operative care. Hospital 2 is a reference center for neonatal, cardiac, neurological, and orthopedic surgeries and childhood cancer treatment. Its neonatal intensive care unit (NICU) has 12 beds.

### Population

The population consisted of nurses and nursing technicians from the neonatal and pediatric intensive care units of the two hospitals, corresponding to a total of 286 professionals (200 from Hospital 1 and 86 from Hospital 2).

### Selection Criteria

The inclusion criteria adopted were: being a nursing professional in a care role at the unit for at least six months. Those who were on vacation or on leave/away during data collection period were excluded.

### Sample Definition

To calculate the sample, the finite population formula was used, estimated at 164 professionals. Sampling was for convenience, with questionnaires being distributed to professionals who met the inclusion criteria. At the end of the data collection period, a total of 100 participants were reached, corresponding to 61% of the estimated sample.

### Data Collection

Two data collection instruments were applied: a sociodemographic and professional questionnaire and the Adverse Events Predisposition Scale – AEPS. The first gathered predictor variables considering the demographic and occupational characteristics of the participants (age, time since training, time working in the service, category, number of jobs, weekly working hours, type of employment relationship, and work shift). AEPS is a Likert-like scale in agreement with two factors that seek to evaluate the attitudes of nurses regarding aspects of the structure and processes that may compromise the quality of nursing care in the Intensive Care Unit (ICU), with the adverse event (AE) as the outcome indicator. It contains 46 items grouped into two dimensions: structure (12 items) and process (34 items)^(9)^.

The scale assesses the level of importance that nurses attribute to aspects of structure and process (ideal level), as well as the perception of the existence of these aspects in their work environment (real level), which can compromise the quality of nursing care in the Intensive Care Unit (ICU)^(9)^.

The structure dimension corresponds to the inputs, physical and financial resources, geographic location, equipment, accessibility and the qualification/specialization of the workforce that enable the provision of the service. The processes dimension deals with the execution of actions through a presupposed set of criteria, rules, standards, procedures and protocols, based on an objective image, to achieve the best assistance^(9)^. The difference between the ideal and real scale represents a score for each assertion.

The questionnaires were self-administered and the professionals were approached at their workplaces, and could return them later, on a date agreed with the researchers.

### Data Analysis and Treatment

Data were analyzed using the statistical software IBM SPSS Statistics 23.0. Descriptive statistics were used with simple frequency and percentage for categorical data, and mean and standard deviation for continuous quantitative data.

To allow the assessment of individual performance in terms of group performance, three percentile ranges were defined. The definition of the values associated with the percentile ranges was then carried out based on the accumulated frequency of this variable. Therefore, values below the 50th percentile represent a low perception of the conditions that may predispose to the occurrence of adverse events; between the 50th and 75th percentiles, an average perception; and, above the 75th percentile, a high perception (Table 1).

**Table 1:** Ranges of the level of perception of AEPS standardization in the structure and process dimensions.

Level	Percentile	Score
Structure dimension		
Low	<50	<1.33
Mean	50 ≤ x ≤75	≥ 1.33 < 2
High	x > 75	≥2
Process dimension		
Low	<50	<1.41
Mean	50 ≤ x ≤75	≥1.41 < 1.91
High	x > 75	≥1.91

Source: Lobão; Menezes (2017).

To compare the difference averages between the ideal and real scales for each item of the dimensions studied, according to the sociodemographic variables, ANOVA was used, followed by the Tukey test; the Student’s t test was used to compare the difference between the ideal and real averages in relation to the nurses’ attitude. A significance level of 0.05 (p < 0.05) was considered.

### Ethical Aspects

The project was approved by the Research Ethics Committee of both institutions (Institution 1 – Opinion No. 2.953.470/2018) and (Institution 2 – Opinion No. 3.023.603/2018). Participants were informed about the nature of the research, receiving clear explanation about it, and signed the Free and Informed Consent Form (FICF).

## RESULTS

A total of 100 nursing workers participated in the study, as approximately 20 workers were on vacation or away and the remainder refused to participate in the research.

The participating nursing team is entirely constituted of women. The mean age was 37.4 ± 9.2 years, the majority were civil servants (68.0%) and unmarried (68.0%), with time since graduation and service time ranging from 1 to 44 years, with averages of 7.9 ± 6.8 and 11 ± 7.9 years, respectively. The time spent working in the neonatal and/or pediatric area is very similar to the length of service, with an average of 7.4 ± 5.9 years. The professionals had a very high weekly workload (52.7 ± 14.9), 22% of them worked between 61 and 96 hours per week, as 49% had two or more jobs.

Regarding the results of the application of AEPS, Table 2 brings together the findings of the participants’ level of perception regarding the factors predisposing to the occurrence of adverse events in the work units. Most professionals demonstrated a low and medium level of perception of structure and process factors (78%), with greater difficulty in the level of perception in the process dimension.

**Table 2:** Distribution of the number of participants according to level of perception of factors predisposing to adverse events in the units – Fortaleza, CE, Brazil, 2019.

Level of perception	Dimension			
	Structure		Process	
	n	%	n	%
Low	43	43.0	51	51.0
Mean	35	35.0	27	27.0
High	22	22.0	22	22.0

Source: Prepared by the author.

Table 3 shows a comparison of the mean scores for the structure and process dimensions, according to the characteristics of the study participants. In the process dimension, the 40–49 age group had the highest average score (1.33) compared to the other age groups (p = 0.040). Regarding the work unit, the average score of hospital 2 NICU (1.52) was higher than that of the other units (p = 0.007). Regarding employment relationships, the average score of 1.31 among cooperative members was higher than that of public servants (p = 0.027). In the structure dimension, no significant differences were observed in the mean scores.

**Table 3:** Comparison of mean scores, according to dimension and demographic/occupational variables – Fortaleza, CE, Brazil, 2019.

Variables	Dimension			
	Structure		Process	
	Mean	SD	Mean	SD
**Position**				
Nurse	1.24	0.72	1.18	0.82
Nursing technician	1.02	0.81	0.94	0.79
p-value*	0.170		0.149	
**Marital status**				
Married	1.24	0.75	1.16	0.95
Not married	1.06	0.79	1.00	0.74
p-value*	0.274		0.350	
**Age**				
22–29	0.98	0.80	0.83	0.87
30–39	1.14	0.63	1.07	0.65
40–49	1.33	0.70	1.35	0.91
50–64	0.78	1.23	0.64	0.76
p-value*	0.192		**0.040**	
**Time since graduation (year)**				
1–4	1.03	0.69	0.95	0.77
5–9	1.00	0.71	0.92	0.82
10–19	1.17	0.60	1.18	0.71
20–44	1.37	1.05	1.26	0.91
p-value*	0.327		0.361	
**Length of service (year)**				
1–4	0.89	0.64	0.82	0.65
5–9	1.26	0.73	1.11	0.86
10–14	1.39	0.73	1.45	0.91
5–44	0.85	1.09	0.83	0.67
p-value*	0.520		0.380	
**Length of service in Neonatology/Pediatrics (years)**				
1–4	0.86	0.76	0.83	0.64
5–9	1.27	0.76	1.16	0.89
10–14	1.33	0.72	1.37	0.86
15–34	1.14	0.83	0.78	0.81
p-value*	0.073		0.061	
**Weekly working hours**				
20–40	0.74	2.56	0.18	0.93
41–60	0.11	3.29	-0.03	1.01
61–96	–1.18	2.85	–0.13	1.04
p-value*	0.084		0.505	
**Unit**				
Pediatric ICU (Hospital 1)	1.29	0.54	1.08	0.61
Post-operative ICU (Hospital 1)	1.02	0.98	0.81	0.61
Medium Risk Neonatal Unit (Hospital 2)	0.94	0.79	0.87	0.82
NICU (Hospital 2)	1.38	0.59	1.52	0.91
p-value*	0.118		**0.007**	
**Type of employment relationship**				
Public Servant	1.06	0.73	0.92	0.80
Cooperative	1.25	0.86	1.31	0.79
p-value*	0.254		**0.027**	

Source: Prepared by the author.

*Student’s t test

**ANOVA test.

Table 4 presents the averages of each of the scale items in the nurses’ perception of the ideal and the real in the structure dimension. The averages of the “ideal” assertions were always above 4.0 and all higher than the “real” assertions (p < 0.0001).

**Table 4:** Comparison of the means of the ideal and real assertions of the AEPS structure dimension – Fortaleza, CE, Brazil, 2019.

Assertive	Type of assertion	Mean	SD	p
1. Adequate lighting for carrying out activities.	Ideal	4.73	0.73	<0.0001
	Real	3.37	1.39	
2. Distribution of beds in a way that favors direct visualization of hospitalized patients.	Ideal	4.80	0.63	<0.0001
	Real	3.53	1.32	
3. Permanent training of the nursing team in the use of biomedical equipment.	Ideal	4.80	0.51	<0.0001
	Real	3.49	1.04	
4. Availability, at the nursing station, of a manual of standards, routines and procedures updated annually.	Ideal	4.72	0.74	<0.0001
	Real	3.58	1.22	
5. Have standardization of solutions and drug dilution.	Ideal	4.89	0.39	<0.0001
	Real	4.39	0.95	
6. Provide catheters, probes, and syringes with devices that prevent incorrect connection or accidental disconnection.	Ideal	4.82	0.53	<0.0001
	Real	3.60	1.31	
7. Have a specific form for reporting adverse events.	Ideal	4.95	0.21	<0.0001
	Real	4.21	1.14	
8. Have a multiparametric monitoring system with follow-up via a central unit at the nursing desk.	Ideal	4.85	0.57	<0.0001
	Real	2.78	1.59	
9. Provide alcohol gel dispensers within beds and at the entrance to the ICU.	Ideal	4.92	0.30	<0.0001
	Real	4.70	0.82	
10. Have equipment of different colors according to the purpose.	Ideal	4.89	0.54	<0.0001
	Real	3.96	1.40	
11. Have a permanent education committee.	Ideal	4.84	0.56	<0.0001
	Real	3.62	1.27	
12. Have a quality care program in the hospital.	Ideal	4.77	0.70	<0.0001
	Real	3.28	1.35	

Source: Prepared by the author.

Student’s t test.

Some aspects stood out as having greater divergence (ideal × real). The items best evaluated in the participants’ real practice involved the presence of alcohol gel dispensers in the unit (4.70), standardization of solutions and drug dilution (4.39), and forms for reporting adverse events (4.21), p < 0.0001 for all items.

The worst rated were: permanent training of the nursing team in the use of biomedical equipment (3.49), adequate lighting (3.37), having a quality of care program in the hospital (3.28) and a multiparametric monitoring system with follow-up through a central unit at the nursing desk (2.78), all with statistical significance (p < 0.0001).

Table 5 presents the averages for each item on the scale according to nurses’ perception of the real and ideal process. The averages of the “ideal” assertions were always above 4.0 and all higher than the “real” assertions (p < 0.0001). There were large discrepancies between real and ideal in the participants’ assessment of most items.

**Table 5:** Comparison of the means of the ideal and real assertions of the AEPS process dimension - Fortaleza, CE, Brazil, 2019.

Assertive	Type of assertion	Mean	SD	p
1. Encourage the nursing team to report the occurrence of adverse events	Ideal	4.84	0.42	<0.0001
	Real	3.51	1.31	
2. Use of the pressure ulcer incidence indicator	Ideal	4.95	0.21	<0.0001
	Real	3.73	1.37	
3. Hand sanitization	Ideal	4.98	0.20	<0.0001
	Real	4.54	0.82	
4. Risk management according to a specific protocol (Ex: RDC-07-2010)	Ideal	4.84	0.58	<0.0001
	Real	3.71	1.26	
5. Medication dispensing system by unit dose and identified by patient	Ideal	4.85	0.59	<0.0001
	Real	3.16	1.57	
6. Use of checklists (bed assembly, shift handover, and pending diagnostic tests)	Ideal	4.82	0.53	<0.0001
	Real	3.67	1.32	
7. Use of at least two identifiers to identify the patient (name and date of birth)	Ideal	4.86	0.69	<0.0001
	Real	4.40	0.99	
8. Frequent patient monitoring analyzing compatibility with data obtained by multiparametric monitors	Ideal	4.92	0.36	<0.0001
	Real	4.10	1.15	
9. Identification of equipment with the solution label and date of change (solutions, sedation, and vasoactive drugs)	Ideal	4.94	0.31	<0.0001
	Real	4.68	0.80	
10. Identification of infusion pumps (solutions, sedation, and vasoactive drugs)	Ideal	4.96	0.24	<0.0001
	Real	4.50	1.04	
11. Use severity index or prognostic index: value that reflects the degree of organ dysfunction of a patient (Ex: APACHE 2)	Ideal	4.84	0.50	<0.0001
	Real	3.18	1.52	
12. Use of evidence-based clinical protocols (e.g. extubation and weaning from MV)	Ideal	4.90	0.38	<0.0001
	Real	3.73	1.43	
13. No use of acronyms that allow for ambiguous interpretation (Ex: IU X IV)	Ideal	4.64	1.04	<0.0001
	Real	3.57	1.35	
14. Use of the accidental extubation incidence indicator	Ideal	4.82	0.57	<0.0001
	Real	3.90	1.27	
15. Use of the bed fall incidence indicator	Ideal	4.85	0.62	<0.0001
	Real	3.73	1.45	
16. Use of the Ramsay Sedation Scale or RASS	Ideal	4.79	0.62	<0.0001
	Real	3.03	1.56	
17. Application of protocols to identify patients with unknown identity, comatose, confused, or under sedation	Ideal	4.85	0.35	<0.0001
	Real	3.25	1.54	
18. Application of SAE steps	Ideal	4.92	0.27	<0.0001
	Real	3.98	1.39	
19. Use of pain as the 5th vital sign	Ideal	4.80	0.68	<0.0001
	Real	3.42	1.53	
20. Use of the fall risk assessment scale (e.g. Morse scale)	Ideal	4.73	0.79	<0.0001
	Real	3.00	1.60	
21. Use of the Glasgow Coma Scale	Ideal	4.63	0.90	<0.0001
	Real	3.25	1.43	
22. Use of a pain intensity assessment scale	Ideal	4.84	0.64	<0.0001
	Real	3.49	1.50	
23. Use of the Braden scale to diagnose the risk of developing pressure ulcers	Ideal	4.87	0.54	<0.0001
	Real	3.53	1.47	
24. Daily clinical discussion of patients’ clinical conditions between nursing assistants and the ICU nursing coordination team	Ideal	4.93	0.29	<0.0001
	Real	3.71	1.40	
25. Performance of systematic position changes every 2 hours in patients with Braden <17	Ideal	4.90	0.46	<0.0001
	Real	4.17	1.18	
26. Use of double-check protocol for medication administration	Ideal	4.87	0.61	<0.0001
	Real	4.37	1.19	
27. Protection of the patient’s skin from excess moisture, dryness, friction, and shear	Ideal	4.85	0.60	<0.0001
	Real	4.34	0.98	
28. Use of insulin therapy protocol	Ideal	4.76	0.76	<0.0001
	Real	3.08	1.14	
29. Use of bed bath protocol for patients on mechanical ventilation	Ideal	4.84	0.64	<0.0001
	Real	3.99	1.37	
30. Use of bed bath protocol for patients on vasoactive drug	Ideal	4.85	0.55	<0.0001
	Real	3.65	1.45	
31. Judicious use of mechanical restraint in cases of psychomotor agitation	Ideal	4.63	0.89	<0.0001
	Real	3.65	1.47	
32. Infusion of blood products exclusively or with 0.9% SF	Ideal	4.89	0.37	<0.0001
	Real	4.19	1.21	
33. Use the non-compliance incidence indicator in medication administration	Ideal	4.87	0.46	<0.0001
	Real	3.93	1.32	
34. Use the hospital infection incidence indicator	Ideal	4.96	0.19	<0.0001
	Real	4.36	0.95	

Source: Prepared by the author.

Student’s t test.

The best evaluated in the real practice of the participants were: identification of equipment with label of solutions and date of exchange (4.68), hand hygiene (4.54), identification of infusion pumps (4.50), use of two identifiers for patient identification (4.40), double-checking protocol in medication administration (4.37), indicator of incidence of hospital infection (4.36), skin protection (4.34), systematic change of position every 2 hours in patients with Braden <17 (4.17), and infusion of blood products exclusively or with 0.9% SF (4.19), all with statistical significance (p < 0.0001).

The worst evaluated items included: using the fall risk assessment scale (3.00); using the Ramsay sedation scale or RASS (3.03); using insulin therapy protocol (3.08); medication dispensing system by unit dose and identified by patient (3.16); using severity index or prognostic index (3.18); applying protocols to identify patients with unknown identity, comatose, confused, or under sedation (3.25); using the Glasgow coma scale (3.25); using pain as the 5th vital sign (3.42); using a pain intensity assessment scale (3.49), all with statistical significance (p < 0.0001).

## DISCUSSION

The participant profile is a portrait of the world’s and Brazilian nursing, especially regarding the predominance of young women, with high workloads and reasonable experience time in the field and area of operation. Other studies carried out with the nursing team have already found similar results regarding the profile presented^(10,11,12)^.

The low level of perception of structure and process factors corroborates the results of the study by the authors of the scale. They found 48.8% of professionals with a low level of perception, 26% with a medium level, and 25.2% with a high level of perception in the structure dimension. Regarding the process dimension, 48% of professionals had a low level of perception, 24.4% had a medium level, and 27.6% had a high level of perception^(9)^, data similar to those of this research. These data indicate that professionals’ perception of these factors is not isolated, but rather a reflection of a broader phenomenon.

The low level of perception in AEPS can represent a major concern in the ICU, since inadequate perceptions can result in attitudes that are harmful to patients. This perception may be compromised due to the fact that healthcare professionals are permeated by multitasking, overloaded with information and, often, with an interruptive workflow in the ICU. Such a stressful work environment reduces the awareness of potential safety risks. It is therefore necessary for management to invest in nurses’ knowledge about preventing adverse events^(13)^.

The low level of perception in the structure dimension, identified in this research, contrasts with the results of a previous study carried out in an adult ICU, in which the nurses’ perception of the presence of structural aspects that can influence the occurrence of adverse events was greater, with agreement of more than 50% in items such as adequate lighting for carrying out activities, distribution of beds in a way that favors direct visualization of patients, availability at the Nursing station of a manual of standards, routines and procedures updated annually, standardization of solutions and drug dilution, alcohol gel dispensers within beds and at the entrance to the ICU, and equipment of different colors according to the purpose, in addition to having its own form for reporting adverse events^(14)^.

A positive perception of these specific items can help workers adopt more careful practices, as a well-structured environment with adequate resources reinforces awareness and attention to risks. The lower perception observed in this research may therefore indicate a lack of knowledge or a lack of adequate training on the importance of these aspects.

There was no statistical significance of the structure domain according to the classification of sociodemographic variables. In the study carried out in an adult ICU, a significant relationship was found between the professionals’ working hours and the structure dimension (p = 0.084)^(14)^. This fact may be influenced by the failure to obtain the estimated sample, which may have reduced its representative power in this aspect. However, the comparison between adult and neonatal/pediatric ICU is a point of divergence that deserves investigation.

The items best evaluated in the participants’ real practice in the structure dimension, with averages above four, were few, involving the presence of alcohol gel dispensers in the unit, standardization of solutions and drug dilution and forms for reporting adverse events. The worst rated items (permanent training of the nursing team in the use of biomedical equipment, adequate lighting, having a quality of care program in the hospital and a multiparametric monitoring system with follow-up through a central unit at the nursing desk) indicate an urgent need for change, being among the items with the greatest discrepancies between the ideal and the real.

These items are the ones deserving the most attention, since a greater divergence between the ideal and the real can be a predictor of an adverse event. The nurse’s perception of this, however, can mobilize prevention factors. The smaller the value of the difference between the ideal and the real, the closer the real scenario is to the ideal.

Regarding the most discrepant items, such as a multiparametric monitoring system with follow-up through a central unit at the nursing desk, as well as bed distribution that favors direct visualization of patients, it seems something simple and standardized, given that ICUs are programmed to have this format. However, some ICUs that are improvised in places not structured for this purpose lack this basic structure. This represents a great risk for critically ill patients, and may result in adverse events (AE) with deaths, in the case of an unidentified cardiopulmonary arrest (CPA), for example.

Other items considered to be quite discrepant were: existence of quality programs and continuing education. The alignment between quality programs in hospitals and ongoing education allows for monitoring and training of staff to improve care. The change in the educational process implemented in a NICU effectively reduced the rate of unmeasurable doses prescribed at discharge from 1 in 4 to 1 in 25, revealing the importance of these educational processes for improving professional practice and patient safety^(15)^.

Regarding lighting, in an observational study in an Australian ICU on the light, sound, and acoustic environment, all bed spaces presented light intensities well below external lighting. Scheduled and automated lighting solutions are available, but staff can nullify this if they are not educated on the essential role light plays in controlling circadian rhythms and supporting sleep. More research is required to determine how improved lighting impacts staff health and performance, and patient experience and outcomes^(16)^.

Regarding devices, an item that also differs between ideal and real, a study carried out in Spain on the acquisition of infusion pumps considers usability as a relevant criterion for selecting devices, highlighting the importance of refining the selection of health technology to improve patient safety^(17)^.

Another item that deserves being highlighted is the availability, at the nursing station, of a manual of standards, routines, and procedures updated annually. Schedules, combined with protocols and checklists, are tools used to assist in carrying out complex routines, increasing safety, reducing costs and optimizing team time^(18)^.

In the process dimension, the best results were: identification of equipment with label of solutions and date of exchange, hand hygiene, identification of infusion pumps, use of two identifiers for patient identification, double-checking protocol in medication administration, indicator of incidence of hospital infection, skin protection, systematic change of position every 2 hours in patients with Braden <17, and infusion of blood products exclusively or with 0.9% SF.

In the study in adult ICU, aspects such as the use of the pressure injury incidence indicator, hand hygiene, use of at least two identifiers to identify the patient, frequent monitoring of the patient analyzing compatibility with the data obtained by multiparametric monitors, identification of equipment with the label of the solutions and date of exchange, and infusion pumps, application of the SAE steps, in addition to the use of evidence-based clinical protocols, insulin therapy, Glasgow coma scale and Braden scale in the diagnosis of risk for the development of decubitus ulcers stand out with a high level of AE perception, presenting some similarities with the results of this research^(14)^.

The worst evaluated items mostly coincided with those with higher divergence between ideal and real: using the fall risk assessment scale; using the Ramsay sedation scale or RASS; using insulin therapy protocol; medication dispensing system by unit dose and identified by patient; using severity index or prognostic index; applying protocols to identify patients with unknown identity, comatose, confused, or under sedation; using the Glasgow coma scale; using pain as the 5th vital sign; using a pain intensity assessment scale.

These items, for the most part, refer to the use of more elaborate scales, indexes or protocols, in addition to items relating to pain. The use of protocols, however, is extremely necessary in the hospital environment, especially in the ICU, whether in the adult or pediatric setting^(19,20,21)^.

An example of how process improvement, using protocols in assessment and conduct, can influence patient safety, was presented in a study on unplanned extubations^(22)^, which have been associated with increased days on the ventilator, risk of infection, cardiopulmonary resuscitation, and use of resuscitation medications. In the research, the authors addressed the steps instituted in a NICU to eliminate this AE and the quality improvement initiative represented a 64% decrease in the total number of extubation events (from 46 to 21) (p < 0.001).

Regarding the importance of pain assessment in the NICU, a cohort study of very premature babies showed that greater exposure to pain early in life was associated with altered maturation of neonatal structural connectivity^(23)^. For this population, analgesia protocols are a challenge, since in very premature neonates, both early exposure to pain and drug analgesia are associated with adverse neurodevelopment and altered brain maturation, with no clear guidelines for the treatment of neonatal pain^(24)^.

The differences in means between real and ideal in the process structure in the variables age, unit and bond were considered statistically significant. In other words, in relation to age, participants aged 40 to 49 demonstrated average perception of process factors, to the detriment of younger and older participants, who demonstrated low perception. This can be justified by the fact that people in the 40–49 age group are considered experienced and generally have other employment relationships, being able to make comparisons with other scenarios closer to the ideal and obtain an average perception of the AE, unlike younger or older people, close to retirement, who are starting or slowing down in the hospital environment.

Regarding the units, the nursing professionals at the NICU of hospital 2 showed a better perception of the process factors, while those at the other hospitals demonstrated a low perception. The NICU is an extremely specialized environment and generally has professionals with specific training, a fact that may have contributed to the better perception.

Moreover, with regard to the employment relationship, cooperative members showed a better perception of procedural factors than public servants. This can be explained by the turnover of members and the possibility of learning about different realities. Public servants often only know the reality of their own work sector. However, studies on the difference in work relationship type have to be carried out regarding the impact on assistance, since it is known that the quality of life of workers without a permanent work relationship is lower than that of those who have a public contract^(25)^. Therefore, the items reported with greater discrepancies require further investigation and planned interventions to prevent AEs.

Nursing professionals realize that adverse events in intensive care are reported spontaneously and that there is a need to train the team in a culture of patient safety and to design an adverse event surveillance system^(26)^.

The results of this study offer important implications for healthcare practice, especially with regard to patient safety and continuous improvement of the quality of care. Nurses’ perception of factors predisposing to adverse events in work units, such as structure and care process, revealed areas for significant improvement. The identification of gaps between the ideal and the real, particularly in relation to aspects such as ongoing training, adequate infrastructure, and safety protocols, points to the need for interventions aimed at the continuous qualification of the team and the optimization of available resources.

Furthermore, the findings on high working hours and working in multiple employment relationships highlight the importance of strategies that promote a balance between the well-being of professionals and patient safety, to ensure quality care with a lower risk of errors. These contributions can support management and training actions, in addition to guiding institutional policies aimed at improving working conditions and reducing adverse events in health units.

As a limitation of the study, it is worth highlighting the use of AEPS only in the adult scenario, making closer comparisons and analyses impossible. Furthermore, the sample calculated for the study was 164 participants, considering a 95% confidence level to ensure the representativeness of the data. However, only 100 professionals actually participated in the research, which may impact the generalization of the results.

## CONCLUSIONS

A predominance of low and medium levels of perception was found regarding the structural and process factors that predispose the occurrence of adverse events in the investigated units. This result may suggest that professionals have an inadequate perception of the quality of the work process, in which failures can become frequent and routine, resulting in negative indicators for the work environment and patient safety.

The result points to the need to look at the worst-rated items in each AEPS dimension, so that managers and leaders can implement improvement measures in the structures and processes of their pediatric and neonatal units.

Given the above, we have an overview of which factors can be prioritized in both institutions to improve the structure and processes capable of providing safety for children and newborns admitted there.

## References

[1] Eldridge N, Wang Y, Metersky M, Eckenrode S, Mathew J, Sonnenfeld N (2022). Trends in adverse event rates in hospitalized patients,
2010–2019. JAMA.

[2] Rocha MS, Gabriel CS, Moura AA, Inácio ALR, Mendonça DF, Bernardes A (2023). Incidência e evitabilidade de eventos adversos no pronto
atendimento: estudo retrospectivo. Acta Paul Enferm.

[3] Babaie M, Nourian M, Atashzadeh-Shoorideh F, Manoochehri H, Nasiri M (2023). Patient safety culture in neonatal intensive care units: a
qualitative content analysis. Front Public Health.

[4] Genna C, Thekkan KR, Raymakers-Janssen PAMA, Gawronski O (2023). Is nurse staffing associated with critical deterioration events
on acute and critical care pediatric wards? A literature
review. Eur J Pediatr.

[5] Zeeshan A, Hashwani Y, Jawwad M, Yousafzai MT, Rehman N, Abbas Q (2023). Safety huddles in paediatric intensive care unit: implementation
and staff perception in a resource limited setting. J Pak Med Assoc.

[6] Ghezaywi Z, Alali H, Kazzaz Y, Ling CM, Esabia J, Murabi I (2024). Targeting zero medication administration errors in the pediatric
intensive care unit: a Quality Improvement project. Intensive Crit Care Nurs.

[7] Lee SE, Dahinten VS, Lee JH (2023). Testing the association between the enabling and enacting factors
of patient safety culture and patient safety: structural equation
modelling. BMC Nurs.

[8] Lugo MS (2023). Notificação de eventos adversos como componente essencial da
segurança do paciente em terapia intensiva. Rev Cubana Enferm.

[9] Lobão WM, Menezes IG (2013). Psychometric analysis of the scale for the predisposition to the
occurrence of adverse events in nursing care provided in
ICUs. Rev Lat Am Enfermagem.

[10] Barros JR, Ramdeen M, Rivera-Sequeiros A, Baima JP, Saad-Hossne R, Alencar RA (2023). Profile of inflammatory bowel disease nurses in
Brazil. Arq Gastroenterol.

[11] Fisher C, Griffith C, Lee H, Smith H, Jones C, Grinke K (2022). Extending the description of the clinical research nursing
workforce. J Res Nurs.

[12] Alanazi S, Wiechula R, Foley D (2022). Followership in nurses working in Saudi Arabian hospitals: a
cross-sectional study. Nurs Forum.

[13] Chen Y, Gong Y (2022). Teamwork and patient safety in intensive care units: challenges
and opportunities. Stud Health Technol Inform.

[14] Nascimento KMB, Silva FM, Santos RA, Santos JCF, Ferreira LC (2020). Predisposição à eventos adversos em Unidade de Terapia
Intensiva. Res Soc Dev.

[15] Zahn SO, Tobison JH, Place AJ, Casey JN, Hess L, Foster T (2020). Impact of a process change on prevalence of prescribed
unmeasurable liquid doses in a neonatal intensive care unit. J Pediatr Pharmacol Ther.

[16] Tronstad O, Flaws D, Patterson S, Holdsworth R, Garcia-Hansen V, Rodriguez Leonard F (2023). Evaluation of the sensory environment in a large tertiary
ICU. Crit Care.

[17] Herrero L, Sánchez-Santiago B, Cano M, Sancibrian R, Ratwani R, Peralta G (2023). Prioritizing patient safety: analysis of the procurement process
of infusion pumps in Spain. Int J Environ Res Public Health.

[18] Taffarel P, Widmer J, Fiore Á, Rodríguez AP, Meregalli C, Jorro Barón F (2023). Impact of the implementation of a sedation and analgesia protocol
in a pediatric intensive care unit. Arch Argent Pediatr.

[19] Arias-Rivera S, Raurell-Torredà M, Fernández-Castillo RJ, Campos-Asensio C, Thuissard-Vasallo IJ, Andreu-Vázquez C (2024). Monitorización de la glucemia en el paciente crítico adulto: tipo
de muestra y método de análisis. Revisión sistemática y metanálisis. Enferm Intensiva.

[20] Kim HJ, Ko RE, Lim SY, Park S, Suh GY, Lee YJ (2024). Sepsis alert systems, mortality, and adherence in emergency
departments: a systematic review and meta-analysis. JAMA Netw Open.

[21] Fuchs A, Koepp G, Huber M, Aebli J, Afshari A, Bonfiglio R (2024). Apnoeic oxygenation during paediatric tracheal intubation: a
systematic review and meta-analysis. Br J Anaesth.

[22] Crezeé KL, DiGeronimo RJ, Rigby MJ, Carter RC, Patel S (2017). Reducing unplanned extubations in the NICU following
implementation of a standardized approach. Respir Care.

[23] Selvanathan T, Ufkes S, Guo T, Chau V, Branson HM, Ibrahim GM (2024). Pain exposure and brain connectivity in preterm
infants. JAMA Netw Open.

[24] Selvanathan T, Zaki P, McLean MA, Au-Young SH, Chau CMY, Chau V (2023). Early-life exposure to analgesia and 18-month neurodevelopmental
outcomes in very preterm infants. Pediatr Res.

[25] Sampaio CL, Aquino JG, Ferreira PC, Ferreira VC, Freitas LH, Almeida NA (2020). Differences between quality of life and occupational coping of
tenured and outsourced nurses. Rev Bras Enferm.

[26] Sarduy-Lugo MV, Rodríguez-Fernández Y, Rodríguez-Medina M, Torres-Cabrera M (2023). Percepción de enfermería sobre la notificación de eventos
adversos en cuidados intensivos pediátricos. Rev Cubana Enferm.

