# Supplementary material for: Attitudes of nursing professionals in adverse events in neonatal and pediatric intensive care
Source: Rev Esc Enferm USP. 2025 Jul 11;59:e20240407. doi: 10.1590/1980-220X-REEUSP-2024-0407en (PMC12250836; doi:10.1590/1980-220X-REEUSP-2024-0407en)
Supplement: Translation

# Translation: Actitudes de los profesionales de enfermería ante eventos adversos en cuidados intensivos neonatales y pediátricos

**DOI:** 10.1590/1980-220X-REEUSP-2024-0407es

**Authors:** Maria Salete de Brito Gomes, Ana Clara de Brito Gomes, Cynthia Lima Sampaio, Paulo César de Almeida, Jênifa Cavalcante dos Santos Santiago, Joselany Áfio Caetano, Roberta Meneses Oliveira

**Affiliations:** 1 Universidade de Fortaleza, Fortaleza, CE, Brasil.; 2 Universidade Federal do Ceará, Faculdade de Farmácia, Odontologia e Enfermagem, Departamento de Enfermagem, Fortaleza, CE, Brasil.; 3 Universidade Federal do Ceará, Departamento de Enfermagem, Programa de Pós-graduação, Fortaleza, CE, Brasil.; 4 Universidade Estadual do Ceará, Fortaleza, CE, Brasil.

**Keywords:** Enfermería, Actitud del Personal de Salud, Unidades de Cuidado Intensivo Neonatal, Seguridad del Paciente, Daño del Paciente

## Abstract

**Objetivo:**

Analizar las actitudes de los profesionales de enfermería hacia los factores estructurales y de proceso que pueden predisponer a eventos adversos en unidades de cuidados intensivos neonatales y pediátricos.

**Método:**

Estudio transversal realizado en dos hospitales públicos del Nordeste de Brasil en 2019. Se aplicó un cuestionario sociodemográfico/profesional y la Escala de Predisposición a Eventos Adversos a 100 profesionales de enfermería. Se utilizaron pruebas ANOVA, *Tukey* y t de Student para comparar los promedios de las escalas ideal y real, según las variables sociodemográficas, adoptando p < 0.05.

**Resultados:**

La mayoría de los profesionales demostraron una percepción baja y media de los factores de estructura y proceso. Se encontró asociación estadísticamente significativa en la dimensión proceso con: grupo etario 40−49 años, unidad neonatal/hospital 2 y vinculación a cooperativa.

**Conclusión:**

Los profesionales de enfermería en unidades de cuidados intensivos neonatales/pediátricos tienen percepción baja y media de los factores predisponentes a eventos adversos, lo que amerita inversión por parte de los líderes para evitar que percepciones inadecuadas resulten en daños a los pacientes.

## INTRODUCCIÓN

Un estudio realizado en los Estados Unidos de América (EEUU) analizó los datos de 244,542 pacientes adultos hospitalizados en 3156 hospitales y observó una reducción de las tasas de eventos adversos entre 2010 y 2019. A pesar de este descenso, las tasas siguen siendo elevadas, con registros de 139 eventos adversos por cada 1000 pacientes con infarto agudo de miocardio, 116 por insuficiencia cardíaca, 119 por neumonía y 130 para los sometidos a procedimientos quirúrgicos mayores^(1)^. En Brasil, la incidencia de acontecimientos adversos fue del 4,4%, en un estudio realizado en la atención de urgencias, todos ellos considerados evitables^(2)^. Estos datos ponen de manifiesto la magnitud del problema y la necesidad de analizar en profundidad la predisposición a los eventos e investigar estrategias que minimicen los riesgos asistenciales, especialmente en los entornos de cuidados intensivos, ya que presentan características peculiares que aumentan los riesgos para los pacientes, como es el caso de las unidades de cuidados intensivos pediátricos y neonatales (UCIP y UCIN). La complejidad de los cuidados prestados a esta clientela, el arsenal terapéutico y la toma de decisiones en las urgencias pediátricas hacen que el entorno sea más propicio para el agotamiento del equipo de enfermería. Ante tal vulnerabilidad, aumenta el número de publicaciones relacionadas con la seguridad de los pacientes sometidos a cuidados intensivos^(3–5)^.

Entre los eventos adversos prevenibles se encuentran los errores de medicación. Los errores de medicación pueden reducirse aplicando varias intervenciones mediante enfoques basados en sistemas humanos y tecnología. Cuando se trata de reducir los errores de medicación en la UCIP y la UCIN, es primordial adoptar un enfoque multidisciplinar^(6)^.

Además del enfoque multidisciplinar, se requiere un enfoque multinivel para reducir los eventos adversos, según un estudio sobre la cultura de la seguridad, que demuestra que los factores facilitadores (actividades a nivel organizativo o de gestión) influyen en los factores de ejecución (acciones del equipo de primera línea) que, a su vez, conducen a resultados en materia de seguridad del paciente. Sin embargo, la naturaleza crítica de la seguridad psicológica entre el personal de enfermería influye en estos resultados^(7)^. De este modo, la perspectiva del profesional y su percepción de la realidad también merecen ser destacadas e investigadas con más detalle.

La no ocurrencia de eventos adversos ha sido un indicador de resultado de la evaluación de la calidad de los cuidados en cuidados intensivos^(8)^. Sin embargo, no sólo la identificación de eventos adversos, sino también la percepción de su riesgo puede orientar el desarrollo de intervenciones correctivas y preventivas, por lo que es fundamental conocer la perspectiva del profesional de enfermería para poder formular abordajes basados en actitudes centradas en la estructura y en el proceso de trabajo.

Teniendo en cuenta lo anterior, el objetivo fue analizar las actitudes de los profesionales de enfermería hacia los factores estructurales y de proceso que pueden predisponer a eventos adversos en UCIN y UCIP.

## MÉTODO

### Diseño del Estudio

Estudio transversal

### Local

El estudio se realizó de enero a abril de 2019, en dos hospitales de referencia para la atención pediátrica y neonatal, ambos especializados en alta complejidad, pertenecientes a la red pública de Ceará. El Hospital 1, con 417 camas, es reconocido nacionalmente por sus especialidades de Cardiología y Neumología. Tiene dos unidades de cuidados intensivos pediátricos (UCIP), con un total de 17 camas, 8 para cuidados postoperatorios y 9 para cuidados preoperatorios. El Hospital 2 es un centro de referencia para cirugías neonatales, cardiacas, neurológicas y ortopédicas y para el tratamiento del cáncer infantil. Su unidad de cuidados intensivos neonatales (UCIN) cuenta con 12 camas.

### Población

La población estaba formada por enfermeras y técnicos de enfermería de las unidades de cuidados intensivos neonatales y pediátricos de los dos hospitales, lo que corresponde a un total de 286 profesionales (200 del Hospital 1 y 86 del Hospital 2).

### Criterios de Selección

Los criterios de inclusión adoptados fueron: ser profesional de enfermería en funciones asistenciales en la unidad durante al menos seis meses. Se excluyeron aquellos que se encontraban de vacaciones o de baja/ausencia durante el periodo de recogida de datos.

### Definición de la Muestra

Para el cálculo de la muestra se utilizó la fórmula de población finita, estimada en 164 profesionales. El muestreo fue por conveniencia, distribuyéndose los cuestionarios a los profesionales que cumplían los criterios de inclusión. Al final del período de recogida de datos, se alcanzó un total de 100 participantes, lo que corresponde al 61% de la muestra estimada.

### Recolección de Datos

Se aplicaron dos instrumentos para recolectar datos: un cuestionario sociodemográfico y profesional y la Escala de Predisposición a Eventos Adversos - (Adverse Events Predisposition Scale - AEPS por su sigla en inglés). El primero recogió variables predictoras considerando las características demográficas y profesionales de los participantes (edad, tiempo desde la formación, tiempo trabajando en el servicio, categoría, número de trabajos, horas de trabajo semanales, tipo de relación laboral y turno de trabajo). La AEPS es una escala tipo Likert de acuerdo con dos factores que pretende evaluar las actitudes de los enfermeros frente a aspectos de la estructura y procesos que pueden comprometer la calidad de los cuidados de enfermería en la Unidad de Cuidados Intensivos (UCI), con el evento adverso (EA) como indicador de resultado. Contiene 46 ítems agrupados en dos dimensiones: estructura (12 ítems) y proceso (34 ítems)^(9)^.

La escala evalúa el nivel de importancia que los enfermeros atribuyen a los aspectos de estructura y proceso (nivel ideal), así como la percepción de la existencia de estos aspectos en su entorno de trabajo (nivel real), que pueden comprometer la calidad de los cuidados de enfermería en la Unidad de Cuidados Intensivos (UCI)^(9)^.

La dimensión estructura corresponde a los insumos, recursos físicos y financieros, localización geográfica, equipamiento, accesibilidad y cualificación/especialización de la fuerza de trabajo que posibilitan la prestación del servicio. La dimensión procesos trata de la ejecución de las acciones a través de un conjunto presupuesto de criterios, reglas, normas, procedimientos y protocolos, basados en una imagen objetiva, para lograr la mejor asistencia^(9)^. La diferencia entre la escala ideal y la real representa una puntuación para cada afirmación.

Los cuestionarios fueron autoadministrados y los profesionales fueron abordados en sus lugares de trabajo, pudiendo devolverlos posteriormente, en una fecha acordada con los investigadores.

### Análisis y Tratamiento de los Datos

Los datos se analizaron con el programa estadístico IBM SPSS Statistics 23,0. Se utilizó estadística descriptiva con frecuencia simple y porcentaje para los datos categóricos, y media y desviación estándar para los datos cuantitativos continuos.

Para permitir la evaluación del rendimiento individual en función del rendimiento del grupo, se definieron tres rangos de percentiles. La definición de los valores asociados a los rangos de percentiles se realizó a continuación a partir de la frecuencia acumulada de esta variable. Así, valores inferiores al percentil 50 representan una percepción baja de las condiciones que pueden predisponer a la ocurrencia de eventos adversos; entre los percentiles 50 y 75, una percepción media; y, por encima del percentil 75, una percepción alta (Tabla 1).

**Tabla 1:** Rangos del nivel de percepción de la normalización de la AEPS en las dimensiones de estructura y proceso.

Nivel	Percentil	Escore
Dimensiones de estructura		
Bajo	<50	<1,33
Medio	50 ≤ x ≤ 75	≥1,33 < 2
Alto	x > 75	≥2
Dimensiones de proceso		
Bajo	<50	<1,41
Medio	50 ≤ x ≤ 75	≥1,41 < 1,91
Alto	x > 75	≥1,91

Fuente: Lobão; Menezes (2017).

Para comparar las medias de diferencia entre las escalas ideal y real para cada ítem de las dimensiones estudiadas, según las variables sociodemográficas, se utilizó el ANOVA, seguido de la prueba de Tukey; para comparar la diferencia entre las medias ideal y real en relación con la actitud de las enfermeras se utilizó el test *t* de Student. Se consideró un nivel de significación de 0,05 (p < 0,05).

### Aspectos Éticos

El proyecto fue aprobado por el Comité de Ética en Investigación de ambas instituciones (Institución 1 - Dictamen nº 2,953,470/2018) e (Institución 2 - Dictamen nº 3,023,603/2018). Los participantes fueron informados sobre la naturaleza de la investigación, recibiendo una explicación clara sobre la misma, y firmaron el Formulario de Consentimiento Libre e Informado (FCLI).

## RESULTADOS

Participaron en el estudio un total de 100 trabajadores de enfermería, ya que aproximadamente 20 trabajadores se encontraban de vacaciones o ausentes y el resto se negó a participar en la investigación.

El equipo de enfermería participante estaba constituido en su totalidad por mujeres. La edad media era de 37,4 ± 9,2 años, la mayoría eran funcionarias (68,0%) y solteras (68,0%), y el tiempo desde la graduación y el tiempo de servicio oscilaban entre 1 y 44 años, con medias de 7,9 ± 6,8 y 11 ± 7,9 años, respectivamente. El tiempo de trabajo en el área neonatal y/o pediátrica es muy similar al tiempo de servicio, con una media de 7,4 ± 5,9 años. Los profesionales tenían una carga de trabajo semanal muy elevada (52,7 ± 14,9), el 22% de ellos trabajaba entre 61 y 96 horas semanales, ya que el 49% tenía dos o más trabajos.

En cuanto a los resultados de la aplicación del AEPS, la Tabla 2 reúne los hallazgos del nivel de percepción de los participantes sobre los factores que predisponen a la ocurrencia de eventos adversos en las unidades de trabajo. La mayoría de los profesionales demostró un nivel bajo y medio de percepción de los factores de estructura y proceso (78%), con mayor dificultad en el nivel de percepción en la dimensión proceso.

**Tabla 2:** Distribución del número de participantes de acuerdo con el nivel de percepción de los factores que predisponen a la ocurrencia de eventos adversos en las unidades. Fortaleza, CE, Brasil, 2019.

Nivel de percepción	Dimensión			
	Estructura		Proceso	
	n	%	n	%
Bajo	43	43,0	51	51,0
Medio	35	35,0	27	27,0
Alto	22	22,0	22	22,0

Fuente: Autores.

La Tabla 3 muestra una comparación de las puntuaciones medias de las dimensiones estructura y proceso, según las características de los participantes en el estudio. En la dimensión de proceso, el grupo de 40–49 años presentó la puntuación media más alta (1,33) en comparación con los demás grupos de edad (p = 0,040). En cuanto a la unidad de trabajo, la puntuación media de la UCIN del hospital 2 (1,52) fue superior a la de las demás unidades (p = 0,007). En cuanto a las relaciones laborales, la puntuación media de los cooperativistas (1,31) fue superior a la de los funcionarios (p = 0,027). En la dimensión estructura, no se observaron diferencias significativas en las puntuaciones medias.

**Tabla 3:** Comparación de las puntuaciones medias, según dimensión y variables demográficas/ocupacionales – Fortaleza, CE, Brasil, 2019.

Variables	Dimensión			
	Estructura		Proceso	
	Media	DE	Media	DE
**Puesto**				
Enfermero/a	1,24	0,72	1,18	0,82
Técnico de enfermería	1,02	0,81	0,94	0,79
Valor de p*	0,170		0,149	
**Estado civil**				
Casado	1,24	0,75	1,16	0,95
No casado	1,06	0,79	1,00	0,74
Valor de p*	0,274		0,350	
**Edad**				
22–29	0,98	0,80	0,83	0,87
30–39	1,14	0,63	1,07	0,65
40–49	1,33	0,70	1,35	0,91
50–64	0,78	1,23	0,64	0,76
Valor de p*	0,192		**0,040**	
**Tiempo desde la graduación (años)**				
1–4	1,03	0,69	0,95	0,77
5–9	1,00	0,71	0,92	0,82
10–19	1,17	0,60	1,18	0,71
20–44	1,37	1,05	1,26	0,91
Valor de p*	0,327		0,361	
**Tiempo de trabajo (años)**				
1–4	0,89	0,64	0,82	0,65
5–9	1,26	0,73	1,11	0,86
10–14	1,39	0,73	1,45	0,91
5–44	0,85	1,09	0,83	0,67
Valor de p*	0,520		0,380	
**Tiempo de trabajo en Neonatología/Pediatría (años)**				
1–4	0,86	0,76	0,83	0,64
5–9	1,27	0,76	1,16	0,89
10–14	1,33	0,72	1,37	0,86
15–34	1,14	0,83	0,78	0,81
Valor de p*	0,073		0,061	
**Horas de trabajo semanales**				
20–40	0,74	2,56	0,18	0,93
41–60	0,11	3,29	-0,03	1,01
61–96	-1,18	2,85	-0,13	1,04
Valor de p*	0,084		0,505	
**Unidad**				
UCI Pediatría (Hospital 1)	1,29	0,54	1,08	0,61
UCI Post-operatoria (Hospital 1)	1,02	0,98	0,81	0,61
Unidad Neonatal de riesgo medio (Hospital 2)	0,94	0,79	0,87	0,82
UCIN (Hospital 2)	1,38	0,59	1,52	0,91
Valor de p*	0,118		**0,007**	
**Tipo de relación de empleo**				
Empleado público	1,06	0,73	0,92	0,80
Cooperativa	1,25	0,86	1,31	0,79
Valor de p*	0,254		**0,027**	

Fuente: autores

*Test t de Student

**ANOVA test. DE: Desvío Estándar.

La Tabla 4 presenta las medias de cada uno de los ítems de la escala en la percepción de las enfermeras sobre lo ideal y lo real en la dimensión estructura. Las medias de las afirmaciones «ideales» fueron siempre superiores a 4,0 y todas superiores a las afirmaciones «reales» (p < 0,0001).

**Tabla 4:** Comparación de las medias de las afirmaciones ideales y reales de la dimensión estructura de la AEPS – Fortaleza, CE, Brasil, 2019.

Afirmación	Tipo de afirmación	Media	DE	p
1. Iluminación adecuada para el desarrollo de las actividades.	Ideal	4,73	0,73	<0,0001
	Real	3,37	1,39	
2. Distribución de camas de forma que se favorezca la visualización directa de los pacientes hospitalizados.	Ideal	4,80	0,63	<0,0001
	Real	3,53	1,32	
3. Formación permanente del equipo de enfermería en el uso de equipos biomédicos.	Ideal	4,80	0,51	<0,0001
	Real	3,49	1,04	
4. Disponibilidad, en el puesto de enfermería, de un manual de normas, rutinas y procedimientos actualizado anualmente.	Ideal	4,72	0,74	<0,0001
	Real	3,58	1,22	
5. Disponer de estandarización de soluciones y dilución de fármacos.	Ideal	4,89	0,39	<0,0001
	Real	4,39	0,95	
6. Proporcionar catéteres, sondas y jeringas con dispositivos que impidan la conexión incorrecta o la desconexión accidental.	Ideal	4,82	0,53	<0,0001
	Real	3,60	1,31	
7. Disponer de un formulario específico para notificar acontecimientos adversos.	Ideal	4,95	0,21	<0,0001
	Real	4,21	1,14	
8. Disponer de un sistema de monitorización multiparamétrica con seguimiento a través de una unidad central en la estación de enfermería.	Ideal	4,85	0,57	<0,0001
	Real	2,78	1,59	
9. Poner dispensadores de alcohol en gel entre las camas y a la entrada de la UCI.	Ideal	4,92	0,30	<0,0001
	Real	4,70	0,82	
10. Disponer de equipos de diferentes colores según la finalidad	Ideal	4,89	0,54	<0,0001
	Real	3,96	1,40	
11. Disponer de un comité de educación permanente	Ideal	4,84	0,56	<0,0001
	Real	3,62	1,27	
12. Disponer de un programa de calidad asistencial en el hospital	Ideal	4,77	0,70	<0,0001
	Real	3,28	1,35	

Fuente: Autores.

Test t de Student. DE: Desvío Estándar.

Algunos aspectos destacaron por presentar mayor divergencia (ideal x real). Los ítems mejor evaluados en la práctica real de los participantes fueron: presencia de dispensadores de alcohol en gel en la unidad (4,70), estandarización de soluciones y dilución de medicamentos (4,39) y formularios para notificación de eventos adversos (4,21), p < 0,0001 para todos los ítems.

Los peor valorados fueron: formación permanente del equipo de enfermería en el uso de equipos biomédicos (3,49), iluminación adecuada (3,37), disponer de un programa de calidad asistencial en el hospital (3,28) y un sistema de monitorización multiparamétrica con seguimiento a través de una unidad central en el mostrador de enfermería (2,78), todos con significación estadística (p < 0,0001).

La Tabla 5 presenta las medias de cada ítem de la escala según la percepción de las enfermeras del proceso real e ideal. Las medias de las afirmaciones «ideales» fueron siempre superiores a 4,0 y todas superiores a las afirmaciones «reales» (p < 0,0001). Hubo grandes discrepancias entre lo real y lo ideal en la evaluación de los participantes de la mayoría de los ítems.

**Tabla 5:** Comparación de las medias de las afirmaciones ideales y reales de la dimensión del proceso AEPS - Fortaleza, CE, Brasil, 2019.

Afirmación	Tipo de afirmación	Media	DE	p
1. Animar al equipo de enfermería a notificar la aparición de acontecimientos adversos	Ideal	4,84	0,42	<0,0001
	Real	3,51	1,31	
2. Utilización del indicador de incidencia de las úlceras por presión	Ideal	4,95	0,21	<0,0001
	Real	3,73	1,37	
3. Desinfección de manos	Ideal	4,98	0,20	<0,0001
	Real	4,54	0,82	
4. Gestión de riesgos según un protocolo específico (Ej: RDC-07-2010)	Ideal	4,84	0,58	<0,0001
	Real	3,71	1,26	
5. Sistema de dispensación de medicamentos por dosis unitaria e identificado por paciente	Ideal	4,85	0,59	<0,0001
	Real	3,16	1,57	
6. Uso de listas de comprobación (montaje de camas, entrega de turnos y pruebas diagnósticas pendientes)	Ideal	4,82	0,53	<0,0001
	Real	3,67	1,32	
7. Uso de al menos dos identificadores para identificar al paciente (nombre y fecha de nacimiento)	Ideal	4,86	0,69	<0,0001
	Real	4,40	0,99	
8. Monitorización frecuente del paciente analizando la compatibilidad con los datos obtenidos por monitores multiparamétricos	Ideal	4,92	0,36	<0,0001
	Real	4,10	1,15	
9. Identificación del equipo con la etiqueta de la solución y la fecha de cambio (soluciones, sedación y fármacos vasoactivos)	Ideal	4,94	0,31	<0,0001
	Real	4,68	0,80	
10. Identificación de bombas de infusión (soluciones, sedación y fármacos vasoactivos)	Ideal	4,96	0,24	<0,0001
	Real	4,50	1,04	
11. Utilizar índice de gravedad o índice pronóstico: valor que refleja el grado de disfunción orgánica de un paciente (Ej: APACHE 2)	Ideal	4,84	0,50	<0,0001
	Real	3,18	1,52	
12. Uso de protocolos clínicos basados en la evidencia (por ejemplo, extubación y destete de la VM)	Ideal	4,90	0,38	<0,0001
	Real	3,73	1,43	
13. No utilizar siglas que permitan una interpretación ambigua (Ej: IU X IV)	Ideal	4,64	1,04	<0,0001
	Real	3,57	1,35	
14. Uso del indicador de incidencia de extubación accidental	Ideal	4,82	0,57	<0,0001
	Real	3,90	1,27	
15. Utilización del indicador de incidencia de caídas de la cama	Ideal	4,85	0,62	<0,0001
	Real	3,73	1,45	
16. Uso de la escala de sedación de Ramsay o RASS	Ideal	4,79	0,62	<0,0001
	Real	3,03	1,56	
17. Aplicación de protocolos para identificar a pacientes de identidad desconocida, comatosos, confusos o bajo sedación	Ideal	4,85	0,35	<0,0001
	Real	3,25	1,54	
18. Aplicación de los pasos SAE	Ideal	4,92	0,27	<0,0001
	Real	3,98	1,39	
19. Utilización del dolor como 5º signo vital	Ideal	4,80	0,68	<0,0001
	Real	3,42	1,53	
20. Utilización de la escala de evaluación del riesgo de caídas (por ejemplo, la escala de Morse)	Ideal	4,73	0,79	<0,0001
	Real	3,00	1,60	
21. Utilización de la escala de coma de Glasgow	Ideal	4,63	0,90	<0,0001
	Real	3,25	1,43	
22. Utilización de una escala de evaluación de la intensidad del dolor	Ideal	4,84	0,64	<0,0001
	Real	3,49	1,50	
23. Utilización de la escala de Braden para diagnosticar el riesgo de desarrollar úlceras por presión	Ideal	4,87	0,54	<0,0001
	Real	3,53	1,47	
24. Discusión clínica diaria de las condiciones clínicas de los pacientes entre los auxiliares de enfermería y el equipo de coordinación de enfermería de la UCI	Ideal	4,93	0,29	<0,0001
	Real	3,71	1,40	
25. Realización de cambios de posición sistemáticos cada 2 horas en pacientes con Braden <17	Ideal	4,90	0,46	<0,0001
	Real	4,17	1,18	
26. Utilización del protocolo de doble control para la administración de medicamentos	Ideal	4,87	0,61	<0,0001
	Real	4,37	1,19	
27. Protección de la piel del paciente contra el exceso de humedad, la sequedad, la fricción y el cizallamiento	Ideal	4,85	0,60	<0,0001
	Real	4,34	0,98	
28. Utilización del protocolo de insulinoterapia	Ideal	4,76	0,76	<0,0001
	Real	3,08	1,14	
29. Uso del protocolo de baño en cama para pacientes con ventilación mecánica	Ideal	4,84	0,64	<0,0001
	Real	3,99	1,37	
30. Uso del protocolo de baño en cama para pacientes con fármacos vasoactivos	Ideal	4,85	0,55	<0,0001
	Real	3,65	1,45	
31. Uso juicioso de la contención mecánica en casos de agitación psicomotriz	Ideal	4,63	0,89	<0,0001
	Real	3,65	1,47	
32. Infusión de productos sanguíneos exclusivamente o con 0,9% SF	Ideal	4,89	0,37	<0,0001
	Real	4,19	1,21	
33. Utilizar el indicador de incidencia de incumplimiento en la administración de medicación	Ideal	4,87	0,46	<0,0001
	Real	3,93	1,32	
34. Utilizar el indicador de incidencia de infecciones hospitalarias	Ideal	4,96	0,19	<0,0001
	Real	4,36	0,95	

Fuente: Autores.

Test t de Student.

Los mejor valorados en la práctica real de los participantes fueron: identificación de equipos con etiqueta de soluciones y fecha de cambio (4,68), higiene de manos (4,54), identificación de bombas de infusión (4,50), uso de dos identificadores para identificación de pacientes (4,40), protocolo de doble comprobación en administración de medicación (4,37), indicador de incidencia de infección hospitalaria (4,36), protección de la piel (4,34), cambio sistemático de posición cada 2 horas en pacientes con Braden <17 (4,17), e infusión de hemoderivados exclusiva o con SF al 0,9% (4,19), todos ellos con significación estadística (p < 0,0001).

Los ítems peor evaluados fueron: uso de la escala de evaluación del riesgo de caídas (3,00); uso de la escala de sedación de Ramsay o RASS (3,03); uso de protocolo de insulinoterapia (3,08); sistema de dispensación de medicación por dosis unitaria e identificada por paciente (3,16); uso de índice de gravedad o índice pronóstico (3,18); aplicación de protocolos para identificar pacientes con identidad desconocida, comatosos, confusos o bajo sedación (3,25); utilización de la escala de coma de Glasgow (3,25); utilización del dolor como 5º signo vital (3,42); utilización de una escala de valoración de la intensidad del dolor (3,49), todos ellos con significación estadística (p < 0,0001).

## DISCUSIÓN

El perfil de los participantes es un retrato de la enfermería mundial y brasileña, especialmente en lo que se refiere al predominio de mujeres jóvenes, con alta carga de trabajo y razonable tiempo de experiencia en el campo y área de actuación. Otros estudios realizados con el equipo de enfermería ya encontraron resultados semejantes en relación al perfil presentado^(10–12)^.

El bajo nivel de percepción de los factores de estructura y proceso corrobora los resultados del estudio de los autores de la escala. Encontraron un 48,8% de profesionales con un nivel de percepción bajo, un 26% con un nivel medio y un 25,2% con un nivel de percepción alto en la dimensión estructura. En cuanto a la dimensión proceso, el 48% de los profesionales tenían un nivel de percepción bajo, el 24,4% un nivel medio y el 27,6% un nivel de percepción alto^(9)^, datos similares a los de esta investigación. Estos datos indican que la percepción de estos factores por parte de los profesionales no es aislada, sino el reflejo de un fenómeno más amplio.

El bajo nivel de percepción en AEPS puede representar una preocupación importante en la UCI, ya que una percepción inadecuada puede dar lugar a actitudes perjudiciales para los pacientes. Esta percepción puede verse comprometida debido al hecho de que los profesionales sanitarios están impregnados de multitarea, sobrecargados de información y, a menudo, con un flujo de trabajo interruptivo en la UCI. Un entorno de trabajo tan estresante reduce la conciencia de los posibles riesgos para la seguridad. Por lo tanto, es necesario que la dirección invierta en los conocimientos del personal de enfermería sobre la prevención de acontecimientos adversos^(13)^.

El bajo nivel de percepción en la dimensión estructura, identificado en esta investigación, contrasta con los resultados de un estudio anterior realizado en una UCI de adultos, en el que la percepción de los enfermeros sobre la presencia de aspectos estructurales que pueden influir en la ocurrencia de eventos adversos fue mayor, con un acuerdo superior al 50% en ítems como iluminación adecuada para la realización de actividades, distribución de las camas de forma que favorezca la visualización directa de los pacientes, disponibilidad en la Enfermería de un manual de normas, rutinas y procedimientos actualizado anualmente, estandarización de soluciones y dilución de medicamentos, dispensadores de alcohol en gel dentro de las camas y en la entrada de la UCI, y equipamiento de diferentes colores según la finalidad, además de disponer de un formulario propio para la comunicación de eventos adversos^(14)^.

Una percepción positiva de estos ítems específicos puede ayudar a los trabajadores a adoptar prácticas más cuidadosas, ya que un ambiente bien estructurado y con recursos adecuados refuerza la conciencia y la atención a los riesgos. Por lo tanto, la menor percepción observada en esta investigación puede indicar un desconocimiento o una falta de formación adecuada sobre la importancia de estos aspectos.

No se encontró significación estadística en el dominio estructura según la clasificación de las variables sociodemográficas. En el estudio realizado en una UCI de adultos, se encontró una relación significativa entre el horario laboral de los profesionales y la dimensión estructura (p = 0,084)^(14)^>. Este hecho puede estar influido por la no obtención de la muestra estimada, lo que puede haber reducido su poder representativo en este aspecto. Sin embargo, la comparación entre UCI de adultos y neonatal/pediátrica es un punto de divergencia que merece ser investigado.

Los ítems mejor valorados en la práctica real de los participantes en la dimensión estructura, con medias por encima de cuatro, fueron pocos, y se referían a la presencia de dispensadores de alcohol en gel en la unidad, estandarización de soluciones y dilución de fármacos y formularios de notificación de eventos adversos. Los ítems peor evaluados (capacitación permanente del equipo de enfermería en el uso de equipos biomédicos, iluminación adecuada, contar con un programa de calidad de la atención en el hospital y un sistema de monitoreo multiparamétrico con seguimiento a través de una unidad central en el escritorio de enfermería) indican una necesidad urgente de cambio, estando entre los ítems con mayores discrepancias entre lo ideal y lo real.

Estos ítems son los que merecen mayor atención, ya que una mayor divergencia entre lo ideal y lo real puede ser un predictor de un evento adverso. Sin embargo, la percepción de esto por parte de la enfermera puede movilizar factores de prevención. Cuanto menor sea el valor de la diferencia entre lo ideal y lo real, más cerca estará el escenario real del ideal.

En cuanto a los ítems más discrepantes, como un sistema de monitorización multiparamétrico con seguimiento a través de una unidad central en la estación de enfermería, así como una distribución de camas que favorezca la visualización directa de los pacientes, parece algo simple y estandarizado, dado que las UCIs están programadas para tener este formato. Sin embargo, algunas UCI improvisadas en lugares no estructurados para ello carecen de esta estructura básica. Esto representa un gran riesgo para los pacientes críticos, pudiendo resultar en eventos adversos (EA) con muertes, en el caso de un paro cardiopulmonar (PCP) no identificado, por ejemplo.

Otros ítems considerados bastante discrepantes fueron: existencia de programas de calidad y formación continuada. El alineamiento entre los programas de calidad en los hospitales y la educación continua permite monitorear y capacitar al personal para mejorar la atención. El cambio en el proceso educativo implantado en una UCIN redujo de forma efectiva la tasa de dosis no medibles prescritas al alta de 1 de cada 4 a 1 de cada 25, lo que revela la importancia de estos procesos educativos para mejorar la práctica profesional y la seguridad del paciente^(15)^.

En cuanto a la iluminación, en un estudio observacional realizado en una UCI australiana sobre el ambiente lumínico, sonoro y acústico, todos los espacios de las camas presentaban intensidades de luz muy por debajo de la iluminación externa. Existen soluciones de iluminación programada y automatizada, pero el personal puede anularlas si no se le educa sobre el papel esencial que desempeña la luz en el control de los ritmos circadianos y el apoyo al sueño. Es necesario seguir investigando para determinar cómo influye la mejora de la iluminación en la salud y el rendimiento del personal, así como en la experiencia y los resultados de los pacientes^(16)^.

En cuanto a los dispositivos, un ítem que también difiere entre lo ideal y lo real, un estudio realizado en España sobre la adquisición de bombas de infusión considera la usabilidad como un criterio relevante para la selección de dispositivos, destacando la importancia de refinar la selección de tecnología sanitaria para mejorar la seguridad del paciente^(17)^.

Otro ítem que merece ser destacado es la disponibilidad, en la estación de enfermería, de un manual de normas, rutinas y procedimientos actualizado anualmente. Los manuales, combinados con protocolos y listas de comprobación, son herramientas utilizadas para ayudar en la realización de rutinas complejas, aumentando la seguridad, reduciendo costes y optimizando el tiempo del equipo^(18)^.

En la dimensión de procesos, los mejores resultados fueron: identificación de equipos con etiqueta de soluciones y fecha de cambio, higiene de manos, identificación de bombas de infusión, uso de dos identificadores para identificación de pacientes, protocolo de doble comprobación en administración de medicación, indicador de incidencia de infección hospitalaria, protección de la piel, cambio sistemático de posición cada 2 horas en pacientes con Braden <17, e infusión de hemoderivados exclusiva o con SF 0,9%.

En el estudio realizado en la UCI de adultos, aspectos como el uso del indicador de incidencia de lesiones por presión, la higiene de manos, el uso de al menos dos identificadores para identificar al paciente, la monitorización frecuente del paciente analizando la compatibilidad con los datos obtenidos por los monitores multiparamétricos, la identificación del equipo con la etiqueta de las soluciones y la fecha de cambio y bombas de infusión, aplicación de los pasos SAE, además del uso de protocolos clínicos basados en la evidencia, destacan con un alto nivel de percepción de EA la insulinoterapia, la escala de coma de Glasgow y la escala de Braden en el diagnóstico de riesgo para el desarrollo de úlceras por decúbito, presentando algunas similitudes con los resultados de esta investigación^(14)^.

Los ítems peor evaluados coincidieron en su mayoría con los de mayor divergencia entre lo ideal y lo real: utilización de la escala de valoración del riesgo de caídas; utilización de la escala de sedación de Ramsay o RASS; utilización del protocolo de insulinoterapia; sistema de dispensación de medicación por dosis unitaria e identificada por paciente; utilización del índice de gravedad o índice pronóstico; aplicación de protocolos para identificar pacientes con identidad desconocida, comatosos, confusos o bajo sedación; utilización de la escala de coma de Glasgow; utilización del dolor como 5º signo vital; utilización de una escala de valoración de la intensidad del dolor.

Estos ítems, en su mayoría, hacen referencia al uso de escalas, índices o protocolos más elaborados, además de los ítems relacionados con el dolor. El uso de protocolos, sin embargo, es extremadamente necesario en el entorno hospitalario, especialmente en la UCI, ya sea en el ámbito adulto o pediátrico^(19−21)^.

Un ejemplo de cómo la mejora de los procesos, mediante el uso de protocolos en la evaluación y la realización, puede influir en la seguridad del paciente, se presentó en un estudio sobre extubaciones no planificadas^(22)^, que se han asociado con un aumento de los días en el respirador, el riesgo de infección, la reanimación cardiopulmonar y el uso de medicamentos de reanimación. En la investigación, los autores abordaron las medidas instituidas en una UCIN para eliminar este EA y la iniciativa de mejora de la calidad representó una disminución del 64% en el número total de eventos de extubación (de 46 a 21) (p < 0,001).

En cuanto a la importancia de la evaluación del dolor en la UCIN, un estudio de cohortes de bebés muy prematuros demostró que una mayor exposición al dolor en etapas tempranas de la vida se asociaba a una alteración de la maduración de la conectividad estructural neonatal^(23)^. Para esta población, los protocolos de analgesia suponen un reto, ya que en los neonatos muy prematuros, tanto la exposición precoz al dolor como la analgesia farmacológica se asocian a un neurodesarrollo adverso y a una maduración cerebral alterada, sin que existan directrices claras para el tratamiento del dolor neonatal^(24)^.

Las diferencias de medias entre real e ideal en la estructura del proceso en las variables edad, unidad y vínculo se consideraron estadísticamente significativas. En otras palabras, en relación con la edad, los participantes de 40 a 49 años demostraron una percepción media de los factores del proceso, en detrimento de los participantes más jóvenes y mayores, que demostraron una percepción baja. Esto se puede justificar por el hecho de que las personas de 40 a 49 años se consideran experimentadas y generalmente tienen otras relaciones laborales, pudiendo hacer comparaciones con otros escenarios más próximos al ideal y obtener una percepción media del EA, a diferencia de las personas más jóvenes o mayores, próximas a la jubilación, que se están iniciando o desacelerando en el ambiente hospitalario.

En cuanto a las unidades, los profesionales de enfermería de la UCIN del hospital 2 mostraron una mejor percepción de los factores de proceso, mientras que los de los otros hospitales mostraron una percepción baja. La UCIN es un entorno extremadamente especializado y generalmente cuenta con profesionales con formación específica, hecho que puede haber contribuido a la mejor percepción.

Por otra parte, en lo que respecta a la relación laboral, los cooperativistas mostraron una mejor percepción de los factores de procedimiento que los funcionarios. Esto puede explicarse por la rotación de los cooperativistas y la posibilidad de conocer realidades diferentes. Los funcionarios públicos a menudo sólo conocen la realidad de su propio sector laboral. Sin embargo, es necesario realizar estudios sobre la diferencia en el tipo de relación laboral en cuanto al impacto en la asistencia, ya que se sabe que la calidad de vida de los trabajadores sin relación laboral permanente es inferior a la de los que tienen un contrato público^(25)^. Por lo tanto, los ítems reportados con mayores discrepancias requieren mayor investigación e intervenciones planificadas para prevenir los EAs.

Los profesionales de enfermería son conscientes de que los eventos adversos en cuidados intensivos se notifican de forma espontánea y que es necesario formar al equipo en una cultura de seguridad del paciente y diseñar un sistema de vigilancia de eventos adversos^(26)^.

Los resultados de este estudio ofrecen importantes implicaciones para la práctica asistencial, especialmente en lo que se refiere a la seguridad del paciente y a la mejora continua de la calidad asistencial. La percepción de las enfermeras sobre los factores que predisponen a la aparición de acontecimientos adversos en las unidades de trabajo, como la estructura y el proceso asistencial, reveló áreas susceptibles de mejoras significativas. La identificación de brechas entre lo ideal y lo real, particularmente en relación a aspectos como formación continuada, infraestructura adecuada y protocolos de seguridad, apunta a la necesidad de intervenciones dirigidas a la cualificación continua del equipo y a la optimización de los recursos disponibles.

Además, los hallazgos sobre la elevada jornada laboral y la pluriactividad ponen de manifiesto la importancia de estrategias que promuevan el equilibrio entre el bienestar de los profesionales y la seguridad de los pacientes, para garantizar una atención de calidad con un menor riesgo de errores. Estos aportes pueden apoyar acciones de gestión y capacitación, además de orientar políticas institucionales dirigidas a mejorar las condiciones de trabajo y reducir los eventos adversos en las unidades de salud.

Como limitación del estudio, cabe destacar la utilización de la AEPS solamente en el escenario adulto, imposibilitando comparaciones y análisis más próximos. Además, la muestra calculada para el estudio fue de 164 participantes, considerando un nivel de confianza del 95% para garantizar la representatividad de los datos. Sin embargo, sólo 100 profesionales participaron realmente en la investigación, lo que puede afectar a la generalización de los resultados.

## CONCLUSIÓN

Se encontró un predominio de los niveles bajo y medio de percepción sobre los factores estructurales y de proceso que predisponen la ocurrencia de eventos adversos en las unidades investigadas. Este resultado puede sugerir que los profesionales tienen una percepción inadecuada de la calidad del proceso de trabajo, en el cual las fallas pueden tornarse frecuentes y rutinarias, resultando en indicadores negativos para el ambiente de trabajo y la seguridad del paciente.

El resultado apunta a la necesidad de observar los ítems peor valorados en cada dimensión de la AEPS, para que los gestores y líderes puedan implementar medidas de mejora en las estructuras y procesos de sus unidades pediátricas y neonatales.

Teniendo en cuenta lo anterior, tenemos una visión general de qué factores pueden priorizarse en ambas instituciones para mejorar la estructura y los procesos capaces de proporcionar seguridad a los niños y recién nacidos allí ingresados.

# Translation: Atitudes de profissionais de enfermagem nos eventos adversos em terapias intensivas neonatal e pediátrica

**DOI:** 10.1590/1980-220X-REEUSP-2024-0407pt

**Authors:** Maria Salete de Brito Gomes, Ana Clara de Brito Gomes, Cynthia Lima Sampaio, Paulo César de Almeida, Jênifa Cavalcante dos Santos Santiago, Joselany Áfio Caetano, Roberta Meneses Oliveira

**Affiliations:** 1 Universidade de Fortaleza, Fortaleza, CE, Brasil.; 2 Universidade Federal do Ceará, Faculdade de Farmácia, Odontologia e Enfermagem, Departamento de Enfermagem, Fortaleza, CE, Brasil.; 3 Universidade Federal do Ceará, Departamento de Enfermagem, Programa de Pós-graduação, Fortaleza, CE, Brasil.; 4 Universidade Estadual do Ceará, Fortaleza, CE, Brasil.

**Keywords:** Enfermagem, Atitude do Pessoal de Saúde, Unidades de Terapia Intensiva Neonatal, Segurança do Paciente, Dano ao paciente

## Abstract

**Objetivo:**

Analisar atitudes de profissionais de enfermagem diante dos fatores de estrutura e processo que podem predispor a eventos adversos em unidades de terapia intensiva neonatal e pediátrica.

**Método:**

Estudo transversal realizado em dois hospitais públicos do Nordeste brasileiro em 2019. Aplicou-se questionário sociodemográfico/profissional e Escala de Predisposição a Eventos Adversos a 100 profissionais de enfermagem. Utilizaram-se os testes ANOVA, *Tukey* e t de *Student* para comparação das médias das escalas ideal e real, segundo as variáveis sociodemográficas, adotando-se p < 0,05.

**Resultados:**

A maioria dos profissionais demonstrou baixa e média percepção dos fatores de estrutura e processo. Constatou-se associação estatística significante na dimensão processo com: faixa etária 40–49 anos, unidade neonatal/hospital 2 e vínculo por cooperativa.

**Conclusão:**

Profissionais de enfermagem de unidades de terapias intensivas neonatal/pediátrica apresentam baixa e média percepção dos fatores predisponentes a eventos adversos, o que merece investimento de lideranças para evitar que percepções inadequadas resultem em dano aos pacientes.

## INTRODUÇÃO

Um estudo realizado nos Estados Unidos da América (EUA) analisou dados de 244.542 pacientes adultos hospitalizados em 3.156 hospitais e identificou uma redução nas taxas de eventos adversos entre 2010 e 2019. Apesar dessa queda, os índices ainda permanecem elevados, com registros de 139 eventos adversos por 1.000 pacientes com infarto agudo do miocárdio, 116 para insuficiência cardíaca, 119 para pneumonia e 130 para aqueles submetidos a procedimentos cirúrgicos importantes^(1)^. No Brasil, a incidência de eventos adversos foi de 4,4%, em estudo realizado em pronto atendimento, sendo todos considerados como evitáveis^(2)^. Esses dados evidenciam a magnitude do problema e a necessidade de aprofundamento na análise da predisposição a eventos e investigação de estratégias que minimizem os riscos assistenciais, especialmente em ambientes de cuidados intensivos, por reunirem características peculiares que aumentam os riscos para os pacientes, como é o caso das unidades de terapia intensiva pediátrica e neonatal (UTIP e UTIN). A complexidade da assistência destinada a essa clientela, o arsenal terapêutico e a tomada de decisão nas urgências pediátricas tornam o ambiente mais propício a um desgaste da equipe de enfermagem. Diante de tal vulnerabilidade, é cada vez mais crescente o número de publicações relacionadas à segurança de pacientes submetidos a cuidados intensivos^(3–5)^.

Entre os eventos adversos preveníveis, estão os erros de medicação. Erros de medicação podem ser diminuídos pela implantação de várias intervenções utilizando abordagens baseadas em sistemas humanos e tecnologia. Quando se trata de reduzir erros de medicação na UTIP e UTIN, uma abordagem multidisciplinar é primordial^(6)^.

Além da abordagem multidisciplinar, é necessário abordagem multinível para redução de eventos adversos, conforme estudo sobre cultura de segurança, que demonstra que os fatores facilitadores (atividades de nível organizacional ou de gestão) influenciam os fatores de execução (ações da equipe da linha de frente) que, por sua vez, levam a resultados de segurança do paciente. Contudo, a natureza crítica da segurança psicológica entre a equipe de enfermagem tem impacto nesse resultado final^(7)^. Dessa maneira, a perspectiva do profissional e sua percepção da realidade também merece destaque e investigação mais detalhada.

A não ocorrência de eventos adversos tem sido um indicador de resultado da avaliação de qualidade do cuidado em terapia intensiva^(8)^. No entanto, não apenas a identificação dos eventos adversos, mas a percepção do risco deles podem orientar o desenvolvimento de intervenções corretivas e preventivas, por isso é primordial entender a perspectiva de olhar do profissional de enfermagem para que abordagens baseadas em atitudes voltadas para estrutura e para o processo de trabalho sejam formuladas.

Diante do exposto, objetivou-se analisar atitudes de profissionais de enfermagem diante dos fatores de estrutura e de processo que podem predispor a eventos adversos em UTIN e UTIP.

## MÉTODO

### Desenho do Estudo

Estudo transversal

### Local

O estudo foi realizado no período de janeiro a abril de 2019, em dois hospitais de referência no atendimento pediátrico e neonatal, ambos especializados em alta complexidade, pertencentes à rede pública do Ceará. O Hospital 1, com 417 leitos, é reconhecido nacionalmente pelas especialidades de Cardiologia e Pneumologia. Possui duas unidades de terapia intensiva pediátrica (UTIP), totalizando 17 leitos, sendo 8 destinados ao pós-operatório e 9 ao pré-operatório. O Hospital 2 é um centro de referência para cirurgias neonatais, cardíacas, neurológicas e ortopédicas e tratamento do câncer infantil. Sua unidade de terapia intensiva neonatal (UTIN) conta com 12 leitos.

### População

A população foi constituída pelos enfermeiros e técnicos de enfermagem das unidades de terapia intensiva neonatal e pediátrica dos dois hospitais, e que correspondiam a um total de 286 profissionais (200 do Hospital 1 e 86 do Hospital 2).

### Critérios de Seleção

Os critérios de inclusão adotados foram: ser profissional de enfermagem em função assistencial na unidade há, pelo menos, seis meses. Foram excluídos os que estavam de férias ou de licença/afastados no período da coleta de dados.

### Definição da Amostra

Para o cálculo da amostra, utilizou-se a fórmula da população finita, sendo estimada em 164 profissionais. A amostragem foi por conveniência, tendo sido distribuídos questionários aos profissionais que atenderam aos critérios de inclusão. Ao final do período de coleta dos dados, chegou-se a um total de 100 participantes, correspondendo a 61% da amostra estimada.

### Coleta de Dados

Aplicaram-se dois instrumentos de coleta dos dados: um questionário sociodemográfico e profissional e a Escala de Predisposição a Eventos Adversos – EPEA. O primeiro reuniu variáveis preditoras contemplando características demográficas e ocupacionais dos participantes (idade, tempo de formação, tempo de atividade no serviço, categoria, quantidade de empregos, carga horária semanal de trabalho, tipo de vínculo empregatício e turno de trabalho). A EPEA é uma escala do tipo *Likert* de concordância com dois fatores que busca avaliar as atitudes dos enfermeiros sobre os aspectos da estrutura e processos que podem comprometer a qualidade do cuidado de enfermagem em Unidade de Terapia Intensiva (UTI), tendo como indicador de resultado o evento adverso (EA). Contém 46 itens agrupados em duas dimensões: estrutura (12 itens) e processo (34 itens)^(9)^.

A escala avalia o nível de importância que os enfermeiros atribuem aos aspectos da estrutura e processo (nível ideal), bem como a percepção sobre a existência desses aspectos no seu ambiente de trabalho (nível real), os quais podem comprometer a qualidade do cuidado de enfermagem em Unidade de Terapia Intensiva (UTI)^(9)^.

A dimensão estrutura corresponde aos insumos, recursos físicos e financeiros, localização geográfica, equipamentos, acessibilidade e a qualificação/especialização da mão de obra que possibilitam a prestação do serviço. A dimensão processos trata da execução de ações mediante um conjunto pressuposto de critérios, regras, padrões, procedimentos e protocolos, a partir de uma imagem objetiva, para alcançar a melhor assistência^(9)^. A diferença entre a escala ideal e a real representa um escore para cada assertiva.

Os questionários foram autoaplicados e os profissionais abordados em seus locais de trabalho, podendo devolver posteriormente, em data acordada com as pesquisadoras.

### Análise e Tratamento dos Dados

Os dados foram analisados no Software estatístico IBM SPSS Statistics 23.0. Utilizou-se estatística descritiva com frequência simples e percentual para os dados categóricos, e média e desvio padrão para os dados quantitativos contínuos.

Para possibilitar a avaliação do desempenho do indivíduo em termos do desempenho do grupo, foram definidas três faixas percentílicas. A definição dos valores associados às faixas percentílicas foi, então, realizada a partir da frequência acumulada dessa variável. Sendo assim, valores abaixo do percentil 50 representam uma baixa percepção sobre as condições que podem predispor à ocorrência de eventos adversos; entre os percentis 50 e 75, uma média percepção; e, acima do percentil 75, uma elevada percepção Tabela 1.

**Tabela 1:** Faixas do nível de percepção da normatização da EPEA nas dimensões estrutura e processo.

Nível	Percentil	Escore
Dimensão estrutura		
Baixo	<50	<1,33
Médio	50 ≤ x ≤ 75	≥ 1,33 < 2
Alto	x > 75	≥2
Dimensão processo		
Baixo	<50	<1,41
Médio	50 ≤ x ≤ 75	≥1,41 < 1,91
Alto	x > 75	≥1,91

Fonte: Lobão; Menezes (2017).

Para comparação das médias da diferença entre as escalas ideal e real para cada item das dimensões estudadas, segundo as variáveis sociodemográficas, foi utilizada a ANOVA, seguida do teste de *Tukey*; o teste t de *Student* foi utilizado para comparação da diferença entre as médias ideal e real em relação à atitude dos enfermeiros. Considerou-se um nível de significância de 0,05 (p < 0,05).

### Aspectos Éticos

O projeto foi aprovado pelo Comitê de Ética em Pesquisa das duas instituições (Instituição 1 – Parecer nº 2.953.470/2018) e (Instituição 2 – Parecer nº 3.023.603/2018). Os participantes foram informados sobre o caráter da pesquisa, recebendo informações claras sobre ela, e assinaram o Termo de Consentimento Livre e Esclarecido (TCLE).

## RESULTADOS

Participaram do estudo 100 trabalhadores de enfermagem, pois cerca de 20 trabalhadores estavam de férias ou afastados e o restante recusou-se a participar da pesquisa.

A equipe de enfermagem participante é em sua totalidade feminina. A média de idade foi de 37,4 ± 9,2 anos, a maioria era funcionária pública (68,0%) e não casada (68,0%), com tempo de formação e tempo de serviço variando de 1 a 44 anos, com médias de 7,9 ± 6,8 e 11 ± 7,9 anos, respectivamente. O tempo de atuação na área neonatal e/ou pediátrica é muito semelhante ao tempo de serviço, com média 7,4 ± 5,9 anos. As profissionais apresentaram carga horária semanal muito elevada (52,7 ± 14,9), 22% delas trabalhavam entre 61 e 96 horas por semana, devido a 49% terem dois ou mais empregos.

No que diz respeito aos resultados da aplicação da EPEA, a Tabela 2 reúne os achados do nível de percepção das participantes quanto aos fatores predisponentes à ocorrência de eventos adversos nas unidades de trabalho. A maioria das profissionais demonstrou nível baixo e médio de percepção dos fatores de estrutura e processo (78%), havendo mais dificuldade do nível de percepção na dimensão processo.

**Tabela 2:** Distribuição do número de participantes segundo nível de percepção dos fatores predisponentes a eventos adversos nas unidades – Fortaleza, CE, Brasil, 2019.

Nível de percepção	Dimensão			
	Estrutura		Processo	
	n	%	n	%
Baixo	43	43,0	51	51,0
Médio	35	35,0	27	27,0
Alto	22	22,0	22	22,0

Fonte: Elaborada pela autora.

A Tabela 3 apresenta a comparação das médias dos escores das dimensões estrutura e processo, de acordo com as características das participantes do estudo. Na dimensão processo, a faixa etária de 40–49 anos apresentou a maior média de escore (1,33) em comparação com as outras faixas etárias (p = 0,040). Em relação à unidade de trabalho, a média de escore da UTIN do hospital 2 (1,52) foi superior à das demais unidades (p = 0,007). Quanto ao vínculo empregatício, a média de escore de 1,31 entre os cooperados foi maior do que a dos servidores públicos (p = 0,027). Já na dimensão estrutura, não foram observadas diferenças significativas nas médias dos escores.

**Tabela 3:** Comparação das médias dos escores, segundo a dimensão e as variáveis demográficas/ocupacionais – Fortaleza, CE, Brasil, 2019.

Variáveis	Dimensão			
	Estrutura		Processo	
	Média	DP	Média	DP
**Cargo**				
Enfermeira	1,24	0,72	1,18	0,82
Técnica de Enfermagem	1,02	0,81	0,94	0,79
p-valor*	0,170		0,149	
**Estado Civil**				
Casada	1,24	0,75	1,16	0,95
Não casada	1,06	0,79	1,00	0,74
p-valor*	0,274		0,350	
**Idade**				
22–29	0,98	0,80	0,83	0,87
30–39	1,14	0,63	1,07	0,65
40–49	1,33	0,70	1,35	0,91
50–64	0,78	1,23	0,64	0,76
p-valor**	0,192		**0,040**	
**Tempo de formação (ano)**				
1–4	1,03	0,69	0,95	0,77
5–9	1,00	0,71	0,92	0,82
10–19	1,17	0,60	1,18	0,71
20–44	1,37	1,05	1,26	0,91
p-valor**	0,327		0,361	
**Tempo de serviço (ano)**				
1–4	0,89	0,64	0,82	0,65
5–9	1,26	0,73	1,11	0,86
10–14	1,39	0,73	1,45	0,91
5–44	0,85	1,09	0,83	0,67
p-valor**	0,520		0,380	
**Tempo de serviço na Neonatologia/pediatria (anos)**				
1–4	0,86	0,76	0,83	0,64
5–9	1,27	0,76	1,16	0,89
10–14	1,33	0,72	1,37	0,86
15–34	1,14	0,83	0,78	0,81
p-valor**	0,073		0,061	
**Carga horária de trabalho semanal**				
20–40	0,74	2,56	0,18	0,93
41–60	0,11	3,29	−0,03	1,01
61–96	−1,18	2,85	−0,13	1,04
p-valor**	0,084		0,505	
**Unidade**				
UTI Pediátrica (Hospital 1)	1,29	0,54	1,08	0,61
UTI Pós-operatória (Hospital 1)	1,02	0,98	0,81	0,61
Unidade Neonatal de Médio Risco (Hospital 2)	0,94	0,79	0,87	0,82
UTIN (Hospital 2)	1,38	0,59	1,52	0,91
p-valor**	0,118		**0,007**	
**Tipo de vínculo empregatício**				
Servidor Público	1,06	0,73	0,92	0,80
Cooperativa	1,25	0,86	1,31	0,79
p-valor*	0,254		**0,027**	

Fonte: Elaborado pela autora.

*Teste t de *Student*

**Teste ANOVA.

A Tabela 4 apresenta as médias de cada um dos itens da escala na percepção dos enfermeiros do ideal e do real na dimensão estrutura. As médias das assertivas “ideal” foram sempre acima de 4,0 e todas maiores do que as assertivas “real” (p < 0,0001).

**Tabela 4:** Comparação das médias das assertivas ideal e real da dimensão estrutura da EPEA – Fortaleza, CE, Brasil, 2019.

Assertiva	Tipo de assertiva	Média	DP	p
1. Iluminação adequada para a execução das atividades.	Ideal	4,73	0,73	<0,0001
	Real	3,37	1,39	
2. Distribuição dos leitos de forma que favoreça a visualização direta dos pacientes internados.	Ideal	4,80	0,63	<0,0001
	Real	3,53	1,32	
3. Capacitação permanente da equipe de enfermagem no uso dos equipamentos biomédicos.	Ideal	4,80	0,51	<0,0001
	Real	3,49	1,04	
4. Disponibilidade no posto de enfermagem de manual de normas, rotinas e procedimentos atualizados anualmente.	Ideal	4,72	0,74	<0,0001
	Real	3,58	1,22	
5. Dispor de padronização de soluções e diluição de drogas.	Ideal	4,89	0,39	<0,0001
	Real	4,39	0,95	
6. Dispor de cateteres, sondas e seringas com dispositivos que previnam conexão incorreta ou desconexão acidental.	Ideal	4,82	0,53	<0,0001
	Real	3,60	1,31	
7. Possuir um formulário próprio para notificação de eventos adversos.	Ideal	4,95	0,21	<0,0001
	Real	4,21	1,14	
8. Dispor de um sistema de monitorização multiparamétrica com acompanhamento por meio de central no balcão de enfermagem.	Ideal	4,85	0,57	<0,0001
	Real	2,78	1,59	
9. Dispor de dispensadores de álcool gel entre os leitos e na entrada da UTI.	Ideal	4,92	0,30	<0,0001
	Real	4,70	0,82	
10. Dispor de equipos de cores diferentes de acordo com a finalidade.	Ideal	4,89	0,54	<0,0001
	Real	3,96	1,40	
11. Dispor de uma comissão de educação permanente.	Ideal	4,84	0,56	<0,0001
	Real	3,62	1,27	
12. Dispor de um programa de qualidade do cuidado no hospital.	Ideal	4,77	0,70	<0,0001
	Real	3,28	1,35	

Fonte: Elaborado pela Autora.

Teste t de Student.

Alguns aspectos destacaram-se como de maior divergência (ideal × real). Os itens mais bem avaliados na prática real das participantes envolveram a presença de dispensadores de álcool gel na unidade (4,70), padronização de soluções e diluição de drogas (4,39) e formulários para notificação de eventos adversos (4,21), p < 0,0001 para todos os itens.

Já os mais mal avaliados foram: capacitação permanente da equipe de enfermagem no uso dos equipamentos biomédicos (3,49), iluminação adequada (3,37), dispor de um programa de qualidade do cuidado no hospital (3,28) e sistema de monitorização multiparamétrica com acompanhamento por meio de central no balcão de enfermagem (2,78), todos com significância estatística (p < 0,0001).

A Tabela 5 apresenta as médias de cada um dos itens da escala na percepção dos enfermeiros do real e do ideal no processo. As médias das assertivas “ideal” foram sempre acima de 4,0 e todas maiores do que as assertivas “real” (p < 0,0001). Houve grandes divergências entre real e ideal na avaliação das participantes na maioria dos itens.

**Tabela 5:** Comparação das médias das assertivas ideal e real da dimensão processo da EPEA – Fortaleza, CE, Brasil, 2019.

Assertiva	Tipo de assertiva	Média	DP	p
1. Estimular a equipe de enfermagem a notificar a ocorrência de eventos adversos	Ideal	4,84	0,42	<0,0001
	Real	3,51	1,31	
2. Utilização do indicador de incidência de úlcera por pressão	Ideal	4,95	0,21	<0,0001
	Real	3,73	1,37	
3. Higienizar as mãos	Ideal	4,98	0,20	<0,0001
	Real	4,54	0,82	
4. Gerenciamento de risco de acordo com um protocolo específico (Ex: RDC-07-2010)	Ideal	4,84	0,58	<0,0001
	Real	3,71	1,26	
5. Sistema de dispensação de medicamentos por dose unitária e identificada por paciente	Ideal	4,85	0,59	<0,0001
	Real	3,16	1,57	
6. Utilizar checklists (montagem de leitos, passagem de plantão e pendência de exames diagnósticos)	Ideal	4,82	0,53	<0,0001
	Real	3,67	1,32	
7. Utilizar no mínimo dois identificadores para identificação do paciente (nome e data de nascimento)	Ideal	4,86	0,69	<0,0001
	Real	4,40	0,99	
8. Monitorização frequente do paciente analisando a compatibilidade com os dados obtidos pelos monitores multiparamétricos	Ideal	4,92	0,36	<0,0001
	Real	4,10	1,15	
9. Identificar equipos com o rótulo das soluções e data de troca (soluções, sedação e drogas vasoativas)	Ideal	4,94	0,31	<0,0001
	Real	4,68	0,80	
10. Identificar bombas de infusão (soluções, sedação e drogas vasoativas)	Ideal	4,96	0,24	<0,0001
	Real	4,50	1,04	
11. Utilizar índice de gravidade ou índice prognóstico: valor que reflete o grau de disfunção orgânica de um paciente (Ex: APACHE 2)	Ideal	4,84	0,50	<0,0001
	Real	3,18	1,52	
12. Utilizar protocolos clínicos baseados em evidência (Ex: extubação e desmame da VM)	Ideal	4,90	0,38	<0,0001
	Real	3,73	1,43	
13. Não utilizar siglas que possibilitem interpretação ambígua (Ex: IU X IV)	Ideal	4,64	1,04	<0,0001
	Real	3,57	1,35	
14. Utilizar o indicador de incidência de extubação acidental	Ideal	4,82	0,57	<0,0001
	Real	3,90	1,27	
15. Utilizar o indicador de incidência de queda do leito	Ideal	4,85	0,62	<0,0001
	Real	3,73	1,45	
16. Utilizar a escala de sedação de Ramsay ou RASS	Ideal	4,79	0,62	<0,0001
	Real	3,03	1,56	
17. Aplicar protocolos para identificação de pacientes com identidade desconhecida, comatosos, confusos ou sob sedação	Ideal	4,85	0,35	<0,0001
	Real	3,25	1,54	
18. Aplicar as etapas da SAE	Ideal	4,92	0,27	<0,0001
	Real	3,98	1,39	
19. Utilizar a dor como 5º sinal vital	Ideal	4,80	0,68	<0,0001
	Real	3,42	1,53	
20. Utilizar a escala de avaliação de risco de queda (Ex: escala de Morse)	Ideal	4,73	0,79	<0,0001
	Real	3,00	1,60	
21. Utilizar a escala de coma de Glasgow	Ideal	4,63	0,90	<0,0001
	Real	3,25	1,43	
22. Utilizar escala de avaliação da intensidade da dor	Ideal	4,84	0,64	<0,0001
	Real	3,49	1,50	
23. Utilizar a escala de Braden no diagnóstico de risco para o desenvolvimento de úlcera por decúbito	Ideal	4,87	0,54	<0,0001
	Real	3,53	1,47	
24. Discussão clínica diária dos quadros clínicos dos pacientes entre os enfermeiros assistenciais e a coordenação de enfermagem da UTI	Ideal	4,93	0,29	<0,0001
	Real	3,71	1,40	
25. Realizar mudança sistemática de decúbito a cada 2 horas nos pacientes com Braden <17	Ideal	4,90	0,46	<0,0001
	Real	4,17	1,18	
26. Utilizar protocolo de dupla-checagem para a administração de medicamentos	Ideal	4,87	0,61	<0,0001
	Real	4,37	1,19	
27. Proteger a pele do paciente do excesso de umidade, ressecamento, fricção e cisalhamento	Ideal	4,85	0,60	<0,0001
	Real	4,34	0,98	
28. Utilizar protocolo de insulinoterapia	Ideal	4,76	0,76	<0,0001
	Real	3,08	1,14	
29.Utilizar protocolo de banho no leito para paciente em ventilação mecânica	Ideal	4,84	0,64	<0,0001
	Real	3,99	1,37	
30.Utilizar protocolo de banho no leito para paciente em uso de droga vasoativa	Ideal	4,85	0,55	<0,0001
	Real	3,65	1,45	
31. Utilização criteriosa de contenção mecânica em caso de agitação psicomotora	Ideal	4,63	0,89	<0,0001
	Real	3,65	1,47	
32. Infusão de hemoderivado em via exclusiva ou com SF 0,9%	Ideal	4,89	0,37	<0,0001
	Real	4,19	1,21	
33. Utilizar o indicador de incidência de não conformidade na administração de medicações	Ideal	4,87	0,46	<0,0001
	Real	3,93	1,32	
34. Utilizar o indicador de incidência de infecção hospitalar	Ideal	4,96	0,19	<0,0001
	Real	4,36	0,95	

**Fonte:** Elaborado pela Autora.

Teste t de *Student*

Os mais bem avaliados na prática real das participantes foram: identificação dos equipos com rótulo das soluções e data das trocas (4,68), higienização das mãos (4,54), identificação das bombas de infusão (4,50), uso de dois identificadores para identificação do paciente (4,40), protocolo de dupla checagem na administração de medicamento (4,37), indicador de incidência de infecção hospitalar (4,36), proteção da pele (4,34), realização de mudança sistemática de decúbito a cada 2 horas nos pacientes com Braden <17 (4,17) e infusão de hemoderivado em via exclusiva ou com o SF 0,9% (4,19), todos com significância estatística (p < 0,0001).

Já os piores itens avaliados incluíram: utilizar a escala de avaliação de risco de queda (3,00); utilizar a escala de sedação de Ramsay ou RASS (3,03); utilizar protocolo de insulinoterapia (3,08); sistema de dispensação de medicamentos por dose unitária e identificada por paciente (3,16); utilizar índice de gravidade ou índice prognóstico (3,18); aplicar protocolos para identificação de pacientes com identidade desconhecida, comatosos, confusos ou sob sedação (3,25); utilizar a escala de coma de Glasgow (3,25); utilizar a dor como 5º sinal vital (3,42); utilizar escala de avaliação da intensidade da dor (3,49), todos com significância estatística (p < 0,0001).

## DISCUSSÃO

O perfil de participantes é um retrato da Enfermagem mundial e brasileira, principalmente quanto à predominância de mulheres jovens, com cargas de trabalho elevadas e tempo razoável de experiência na unidade e na área de atuação. Outras pesquisas realizadas com a equipe de enfermagem já encontraram resultados semelhantes quanto ao perfil apresentado^(10–12)^.

O baixo nível de percepção dos fatores de estrutura e processo corroboram os resultados do estudo dos autores da escala. Eles encontraram 48,8% dos profissionais com baixo nível de percepção, 26% com médio nível e 25,2% com alto nível de percepção na dimensão estrutura. Quanto à dimensão processo, 48% dos profissionais possuíam baixo nível de percepção, 24,4% médio nível e 27,6% alto nível de percepção^(9)^, dados semelhantes aos desta pesquisa. Esses dados indicam que a percepção dos profissionais sobre esses fatores não é isolada, mas sim um reflexo de um fenômeno mais amplo.

O baixo nível de percepção na EPEA pode representar uma grande preocupação na UTI, visto que percepções inadequadas podem resultar em atitudes com danos aos pacientes. Essa percepção pode estar comprometida devido ao fato de os profissionais de saúde serem permeados de multitarefas, sobrecarregados de informações e, frequentemente, com um fluxo de trabalho interruptivo em UTI. Um ambiente de trabalho tão estressante reduz sua conscientização sobre potenciais riscos de segurança. É necessário, portanto, investimento da gestão no conhecimento de enfermeiros sobre prevenção de eventos adversos^(13)^.

O baixo nível de percepção na dimensão estrutura, identificado nesta pesquisa, contrasta com os resultados de um estudo anterior realizado em UTI adulta, no qual a percepção dos enfermeiros sobre a presença de aspectos estruturais que podem influenciar na ocorrência de eventos adversos foi maior, com concordância de mais de 50% em itens como iluminação adequada para a execução das atividades, distribuição dos leitos de forma que favoreça a visualização direta dos pacientes, disponibilidade no posto de Enfermagem de manual de normas, rotinas e procedimentos atualizados anualmente, de padronização de soluções e diluição de drogas, de dispensadores de álcool gel entre os leitos e na entrada da UTI e de equipos de cores diferentes de acordo com a finalidade, além de possuir formulário próprio para notificação de eventos adversos^(14)^.

A percepção positiva sobre esses itens específicos pode contribuir para que os trabalhadores adotem práticas mais cuidadosas, pois um ambiente bem estruturado e com recursos adequados reforça a conscientização e a atenção para os riscos. A percepção mais baixa observada nesta pesquisa pode, portanto, indicar uma falta de conhecimento ou uma carência de treinamento adequada sobre a importância desses aspectos.

Não houve significância estatística do domínio estrutura de acordo com a classificação das variáveis sociodemográficas. Já no estudo realizado em UTI adulta, constatou-se uma relação significativa entre a jornada de trabalho dos profissionais e a dimensão estrutura (p = 0,084)^(14)^. Tal fato pode ser influenciado pela não obtenção da amostra estimada, podendo ter diminuído o seu poder de representatividade neste aspecto. Contudo, a comparação entre UTI adulta e neonatal/pediátrica é um ponto de divergência que merece investigação.

Os itens mais bem avaliados na prática real das participantes na dimensão estrutura, com médias acima de quatro, foram poucos, envolvendo presença de dispensadores de álcool gel na unidade, padronização de soluções e diluição de drogas e formulários para notificação de eventos adversos. Os piores avaliados (capacitação permanente da equipe de enfermagem no uso dos equipamentos biomédicos, iluminação adequada, dispor de um programa de qualidade do cuidado no hospital e sistema de monitorização multiparamétrica com acompanhamento por meio de central no balcão de enfermagem) indicam uma necessidade urgente de mudança, estando dentre os itens com maiores divergências entre o ideal e o real.

Esses itens são os que mais merecem atenção, visto que uma maior divergência entre o ideal e o real pode ser um preditor do evento adverso. A percepção do enfermeiro sobre isso, contudo, pode mobilizar fatores de prevenção. E quanto menor o valor da diferença entre o ideal e o real, mais próximo o cenário real está do ideal.

Em relação aos itens mais discrepantes, como sistema de monitorização multiparamétrica com acompanhamento por meio de central no balcão de enfermagem, assim como distribuição dos leitos que favoreça a visualização direta dos pacientes, parece algo simples e padronizado, visto que as UTIs são programadas para terem esse formato. No entanto, algumas UTIs que são improvisadas em locais que não foram estruturados com essa finalidade carecem dessa estrutura básica. Isso representa um grande risco para pacientes graves, podendo resultar em eventos adversos (EA) com óbitos, no caso de uma parada cardiorrespiratória (PCR) não identificada, a exemplo.

Outros itens tidos como bastante discrepantes foram: existência de programas de qualidade e educação permanente. O alinhamento entre programas de qualidade nos hospitais e educação permanente permite monitoramento e capacitação da equipe para melhoria na assistência. A mudança do processo educacional implementada numa UTIN reduziu efetivamente a taxa de doses não mensuráveis prescritas na alta de 1 em 4 para 1 em 25, revelando a importância destes processos educativos para a melhoria da prática profissional e para a segurança do paciente^(15)^.

Quanto à iluminação, em estudo observacional em UTI australiana sobre o ambiente de luz, som e acústica, todos os espaços do leito apresentaram intensidades de luz bem abaixo da iluminação externa. Soluções de iluminação programadas e automatizadas estão disponíveis, mas a equipe pode anular isso se não for educada sobre o papel essencial que a luz desempenha no controle dos ritmos circadianos e no suporte ao sono. Mais pesquisas são necessárias para determinar como a iluminação melhorada impacta na saúde e no desempenho da equipe, e na experiência e nos resultados dos pacientes^(16)^.

Em relação aos dispositivos, item também discrepante entre ideal e real, estudo realizado na Espanha sobre a aquisição de bombas de infusão considera a usabilidade como um critério relevante para seleção dos dispositivos, sendo apontada a importância de refinar a seleção de tecnologia de saúde para melhorar a segurança do paciente^(17)^.

Outro item que merece destaque em divergência é o de disponibilidade no posto de enfermagem de manual de normas, rotinas e procedimentos atualizados anualmente. As escalas, aliadas aos protocolos e checklists, são ferramentas utilizadas para auxiliar na realização de rotinas complexas, aumentando a segurança, diminuindo gastos e otimizando o tempo da equipe^(18)^.

Na dimensão processo, os melhores resultados foram: identificação dos equipos com rótulo das soluções e data das trocas, higienização das mãos, identificação das bombas de infusão, uso de dois identificadores para identificação do paciente, protocolo de dupla checagem na administração de medicamento, indicador de incidência de infecção hospitalar, proteção da pele, realização de mudança sistemática de decúbito a cada 2 horas nos pacientes com Braden <17 e infusão de hemoderivado em via exclusiva ou com o SF 0,9%.

Já no estudo em UTI adulta, destacam-se, com alto nível de percepção de EA, aspectos como utilização do indicador de incidência de lesão por pressão, higienização das mãos, utilização de no mínimo dois identificadores para identificação do paciente, monitorização frequente do paciente analisando a compatibilidade com os dados obtidos pelos monitores multiparamétricos, identificação dos equipos com o rótulo das soluções e data de troca, e as bombas de infusão, aplicação das etapas da SAE, além da utilização de protocolos clínicos baseados em evidência, de insulinoterapia, escala de coma de Glasgow e escala de Braden no diagnóstico de risco para o desenvolvimento de úlcera por decúbito, apresentando algumas semelhanças com os resultados desta pesquisa^(14)^.

Os piores itens avaliados coincidiram, em sua maioria, com os de maiores divergências entre ideal e real: utilizar a escala de avaliação de risco de queda; utilizar a escala de sedação de Ramsay ou RASS; utilizar protocolo de insulinoterapia; sistema de dispensação de medicamentos por dose unitária e identificada por paciente; utilizar índice de gravidade ou índice prognóstico; aplicar protocolos para identificação de pacientes com identidade desconhecida, comatosos, confusos ou sob sedação; utilizar a escala de coma de Glasgow; utilizar a dor como 5º sinal vital; utilizar escala de avaliação da intensidade da dor.

Esses itens, em sua maioria, referem-se à utilização de escalas, índices ou protocolos mais elaborados, além de itens referentes à dor. A utilização de protocolos, entretanto, é extremamente necessária no ambiente hospitalar, especialmente na UTI, seja no cenário adulto ou pediátrico^(19−21)^.

Um exemplo de como a melhoria dos processos, utilizando protocolos na avaliação e conduta, pode influenciar a segurança do paciente, foi apresentado em estudo sobre extubações não planejadas^(22)^, as quais têm sido associadas ao aumento de dias no ventilador, risco de infecção, ressuscitação cardiopulmonar e uso de medicamentos para ressuscitação. Na pesquisa, os autores abordaram as etapas instituídas em uma UTIN para eliminar este EA e a iniciativa de melhoria da qualidade representou diminuição de 64% no total de eventos de extubação (de 46 para 21) (p < 0,001).

Quanto à importância da avaliação da dor em UTIN, um estudo de coorte de bebês muito prematuros evidenciou que maior exposição à dor no início da vida foi associada à maturação alterada da conectividade estrutural neonatal^(23)^. Para essa população, protocolos para analgesia constituem um desafio, pois em neonatos muito prematuros, tanto a exposição precoce à dor quanto a analgesia medicamentosa estão associadas ao neurodesenvolvimento adverso e à maturação cerebral alterada, sem diretrizes claras para o tratamento da dor neonatal^(24)^.

Foram considerados estatisticamente significantes as diferenças de médias entre real e ideal na estrutura processo nas variáveis idade, unidade e vínculo. Ou seja, em relação à idade, as participantes de 40 a 49 anos manifestaram média percepção dos fatores de processo em detrimento das mais jovens e mais velhas, que demonstraram baixa percepção. Isso pode ser justificado pelo fato de que pessoas na faixa etária de 40–49 anos são consideradas experientes e geralmente possuem outros vínculos empregatícios, podendo realizar comparações com outros cenários mais próximos do ideal e obter uma média percepção do EA, diferentemente dos mais jovens ou mais velhos, próximos da aposentadoria, que estão iniciando ou desacelerando do ritmo no ambiente hospitalar.

Quanto às unidades, as profissionais de enfermagem da UTIN do hospital 2 mostraram melhor percepção dos fatores de processo, enquanto que as dos demais hospitais demonstraram baixa percepção. A UTIN é um ambiente extremamente especializado e geralmente conta com profissionais com formações específicas, fato que pode ter contribuído para a melhor percepção.

E, no que diz respeito ao vínculo empregatício, os cooperados mostraram melhor percepção dos fatores processuais do que os servidores públicos. Isso pode ser explicado pela rotatividade dos cooperados e possibilidade de conhecimento de diferentes realidades. O servidor público, muitas vezes, só conhece a realidade do mesmo setor de trabalho. No entanto, estudos sobre a diferença de vínculos precisam ser realizados em relação ao impacto na assistência, uma vez que é sabido que a qualidade de vida do trabalhador sem vínculo é menor do que a do concursado^(25)^. Portanto, os itens relatados com maiores divergências precisam de maiores investigações e intervenções programadas para a prevenção dos EA.

Profissionais de enfermagem percebem que os eventos adversos em terapia intensiva são notificados espontaneamente e que há necessidade de capacitar a equipe em uma cultura de segurança do paciente e desenhar um sistema de vigilância de eventos adversos^(26)^.

Os resultados deste estudo oferecem importantes implicações para a prática assistencial, especialmente no que se refere à segurança do paciente e à melhoria contínua da qualidade da assistência. A percepção das enfermeiras sobre os fatores predisponentes a eventos adversos nas unidades de trabalho, como a estrutura e o processo assistencial, revelou áreas de melhoria significativa. A identificação de lacunas entre o ideal e o real, particularmente em relação a aspectos como capacitação permanente, infraestrutura adequada e protocolos de segurança, aponta para a necessidade de intervenções direcionadas à qualificação contínua da equipe e à otimização dos recursos disponíveis.

Além disso, os achados sobre a carga horária de trabalho elevada e a atuação em múltiplos vínculos empregatícios ressaltam a importância de estratégias que promovam o equilíbrio entre o bem-estar dos profissionais e a segurança do paciente, para garantir uma assistência de qualidade e com menor risco de erros. Essas contribuições podem subsidiar ações de gestão e de formação, além de orientar políticas institucionais voltadas para a melhoria das condições de trabalho e a redução dos eventos adversos nas unidades de saúde.

Como limitação do estudo, ressalta-se a utilização da EPEA apenas no cenário adulto, impossibilitando comparações e análises mais aproximadas. Além disso, a amostra calculada para o estudo foi de 164 participantes, considerando um nível de confiança de 95% para garantir a representatividade dos dados. No entanto, apenas 100 profissionais participaram efetivamente da pesquisa, o que pode impactar a generalização dos resultados.

## CONCLUSÕES

Encontrou-se predominância de baixo e médio nível de percepção quanto aos fatores de estrutura e processo que predispõem a ocorrência de eventos adversos nas unidades investigadas. Tal resultado pode sugerir que os profissionais têm uma percepção inadequada quanto à qualidade no processo de trabalho, em que as falhas podem se tornar frequentes e rotineiras, resultando em indicadores negativos para o ambiente de trabalho e a segurança do paciente.

O resultado aponta para a necessidade de lançar um olhar para os itens mais mal avaliados em cada dimensão da EPEA, para que gestores e líderes consigam implementar medidas de melhoria nas estruturas e nos processos de suas unidades pediátricas e neonatais.

Diante do exposto, tem-se um panorama sobre que fatores podem ser priorizados nas duas instituições para melhorar estrutura e processos capazes de proporcionar a segurança de crianças e neonatos ali internados.
